# Inter-compound and Intra-compound Global Sensitivity Analysis of a Physiological Model for Pulmonary Absorption of Inhaled Compounds

**DOI:** 10.1208/s12248-020-00499-0

**Published:** 2020-08-30

**Authors:** Nicola Melillo, Silvia Grandoni, Nicola Cesari, Giandomenico Brogin, Paola Puccini, Paolo Magni

**Affiliations:** 1 Laboratory of Bioinformatics, Mathematical Modelling and Synthetic Biology, Department of Electrical, Computer and Biomedical Engineering, Università degli Studi di Pavia, Via Ferrata 5, I-27100 Pavia, Italy; 2 Pharmacokinetics, Biochemistry and Metabolism Department, Chiesi Farmaceutici S.p.A., Parma, Italy

**Keywords:** global sensitivity analysis, *inter-compound*, *intra-compound*, pulmonary absorption model, PBPK, model building, model assessment

## Abstract

In recent years, global sensitivity analysis (GSA) has gained interest in physiologically based pharmacokinetics (PBPK) modelling and simulation from pharmaceutical industry, regulatory authorities, and academia. With the case study of an in-house PBPK model for inhaled compounds in rats, the aim of this work is to show how GSA can contribute in PBPK model development and daily use. We identified two types of GSA that differ in the aims and, thus, in the parameter variability: *inter-compound* and *intra-compound* GSA. The *inter-compound* GSA aims to understand which are the parameters that mostly influence the variability of the metrics of interest in the whole space of the drugs’ properties, and thus, it is useful during the model development. On the other hand, the *intra-compound* GSA aims to highlight how much the uncertainty associated with the parameters of a given drug impacts the uncertainty in the model prediction and so, it is useful during routine PBPK use. In this work, *inter-compound* GSA highlighted that dissolution- and formulation-related parameters were mostly important for the prediction of the fraction absorbed, while the permeability is the most important parameter for lung AUC and MRT. *Intra-compound* GSA highlighted that, for all the considered compounds, the permeability was one of the most important parameters for lung AUC, MRT and plasma MRT, while the extraction ratio and the dose for the plasma AUC. GSA is a crucial instrument for the quality assessment of model-based inference; for this reason, we suggest its use during both PBPK model development and use.

**Electronic supplementary material:**

The online version of this article (10.1208/s12248-020-00499-0) contains supplementary material, which is available to authorized users.

## INTRODUCTION

Due to the opportunity of directly targeting the biophase of interest, the inhalation route has been considered a convenient way of drug administration for local treatment of lung-specific diseases, such as asthma and chronic obstructive pulmonary disease (COPD). This route allows the administration of drugs at lower dosages, minimizing potential side effects driven by high systemic exposures. Topically active compounds for lung diseases have normally an adequate, and generally sustained, lung residence time (1–3). However, efforts have to be placed in the optimization of drug lung disposition looking for an optimal lung retention. In fact, an increased residence time in the airways could potentially translate into the risk of drug removal from the lung due to mucociliary clearance or into the risk of unsafe drug accumulation in pulmonary tissues. For this reason, it is necessary to maintain an appropriate balance between lung retention and absorption by the modulation of the interplay of some key properties, such as solubility, permeability and lung tissue binding (1,3).

Nowadays, administration by inhalation to rodents is still an important step in preclinical development of new drugs designed for the inhalation route (1)**.** A mechanistic model able to predict pharmacokinetic quantities of interest after inhalation of new compounds in preclinical species could be extremely beneficial during drug discovery (4), for example for prioritizing compounds before animal experiments or for preclinical to clinical translation.

For these reasons, in a previous work we developed a physiologically based pharmacokinetic (PBPK) model for inhaled drugs (5). This model was used to predict the compounds lung disposition in preclinical species (e.g. rodents), starting from physiological and *in vitro* parameters, such as mucociliary clearance rate, drug solubility and permeability.

In recent years sensitivity analysis, including global sensitivity analysis (GSA), has gained interest in PBPK modelling and simulation from both industry and academia (6–11). In a recent work, sensitivity, uncertainty and variability analyses were highlighted as tools able to improve the confidence in the context of inhalation PBPK modelling (12). Moreover, regulatory agencies highlighted that a sensitivity analysis should be performed during the process of development and refinement of PBPK models (13,14). Sensitivity analysis could be defined as “The study of how uncertainty in the output of a model (numerical or otherwise) can be apportioned to different sources of uncertainty in the model input*”* (15). GSA differs with respect to other types of sensitivity analysis, such as the local one, as it performs a multivariate variation of all the considered input parameters taken in their whole range of variation (16).

Like all the mechanistic models, physiological models have both uncertainty and variability in the model (input) parameters. Uncertainty refers to an incomplete understanding of the system, lack of data or error in the measurement/estimation of certain parameters. Variability instead refers to the inherent heterogeneity of the system properties or parameters, for example among subjects, experiments, or drugs. It is possible to reduce the uncertainty by performing better experiments and collect more data. Instead, it is impossible to reduce the variability (given a certain context); however, it could be better characterized (https://www.epa.gov/expobox/uncertainty-and-variability). Given the uncertainty (or variability) in the model parameters, it follows that also the predicted model outputs are uncertain (or variable) too (16). In our case, for example, the rat weight could be seen as a variable input parameter, while the values of the active and passive permeability across lung tissues as uncertain parameters. Pulmonary and plasma AUC are examples of pharmacokinetic output metrics of interest, whose uncertainties are driven by those of the model parameters.

Sensitivity analysis helps to get insights into the model behaviour as a function of the parameter variation (16). By performing this type of analysis, it is in fact possible to assess how much each parameter, with its variation, impacts the variation of the model outputs (16). Consequently, sensitivity analysis could be a valuable instrument to help in understanding if the model behaves as expected or what the parameters are that need to be more precisely known to allow reliable model predictions.

In this context, the aim of our work was to understand how GSA can be integrated in the process of PBPK model development and use. This was done within the case study of the in-house PBPK model for inhaled compounds in rats. Among all GSA methods, we choose the variance-based method because of its ability to detect interaction effects and to handle nonlinear and nonmonotonic relationships between the parameters and the model output (16–18).

In this work, we performed two types of GSA that differ in the aim and thus in the considered drug-specific parameter variability range: *inter-compound* and *intra-compound GSA*. *Inter-compound* GSA resembles the analysis done in Melillo *et al.* (6) for intestinal absorption models. Each of the drug-related model parameters was considered variable in a range given by the minimum and the maximum value of the considered set of compounds. Thus, *inter-compound* GSA mainly focalize on the “between-drug” parameter variability and it is useful to understand the model behaviour in the subspace of all the considered compounds. The aim of this analysis is to understand what the key parameters are that mostly explain the differences in the model predictions among drugs. On the other hand, *intra-compound* GSA is focused on the parameter uncertainties related to a specific compound. This analysis has the purpose of finding the most important parameters that, with their uncertainty, mostly cause the model output uncertainty. In this work, *intra-compound* GSA was performed on three representative compounds belonging to the Chiesi Farmaceutici portfolio (namely A, B and C). For each compound, drug-related parameters were considered to vary in a range representing the parameter uncertainty.

## MATERIALS AND METHODS

### Variance-Based GSA

Let us consider the generic model

1 $$ Y=f\left(\boldsymbol{X}\right) $$

with *Y* a scalar model output (e.g. plasma drug AUC), *f* the input-output relationship and ***X*** the vector of the *k* uncorrelated input parameters. GSA methods consider each *X*_*i*_, *i* = 1…*k*, as a random variable, with associated a probability distribution (16,17). Thus, *Y* is a random variable too and can be obtained through model evaluation after sampling from the joint probability distribution of ***X***. For each *X*_*i*_, variance-based GSA derives two sensitivity indices from the decomposition of the variance (*V*) of *Y*, the so-called main (or first order) effect (*S*_*i*_) and total effect (*S*_*T*,*i*_), in Eqs. (2) and (3), respectively (16,17).

2 $$ {S}_i=\frac{V_{X_i}\left({E}_{{\boldsymbol{X}}_{\sim i}}\left(Y|{X}_i\right)\right)}{V(Y)} $$

3 $$ {S}_{T,i}=\frac{E_{{\mathbf{X}}_{\sim i}}\left({V}_{X_i}\left(Y|{\boldsymbol{X}}_{\sim i}\right)\right)}{V(Y)} $$

*E* is the expected value and ***X***_~*i*_ is the vector containing all the parameters, except *X*_*i*_. Both *S*_*i*_ and *S*_*T*, *i*_ are always included in [0, 1]. *S*_*i*_ is related with the part of *V*(*Y*) explained by the variation of *X*_*i*_ taken alone, and *S*_*T*, *i*_ is the sum of *S*_*i*_ with the interaction effects of *X*_*i*_ with all the other inputs. Interaction effects can arise when more than one parameter varies at the same time. The relationship *S*_*i*_ ≤ *S*_*T*, *i*_ is always valid. The higher *S*_*i*_ and *S*_*T*, *i*_ are, the more the variation of *X*_*i*_ explains *V*(*Y*) and so, the more *X*_*i*_ is considered important. Instead, *S*_*T*, *i*_ = 0 means that *X*_*i*_ is not influent on *V*(*Y*). The difference *S*_*T*, *i*_ − *S*_*i*_ gives information about the extent of interaction effect involving the *i*th parameter (16,17).

### Pulmonary Absorption Model

**Fig. 1:**
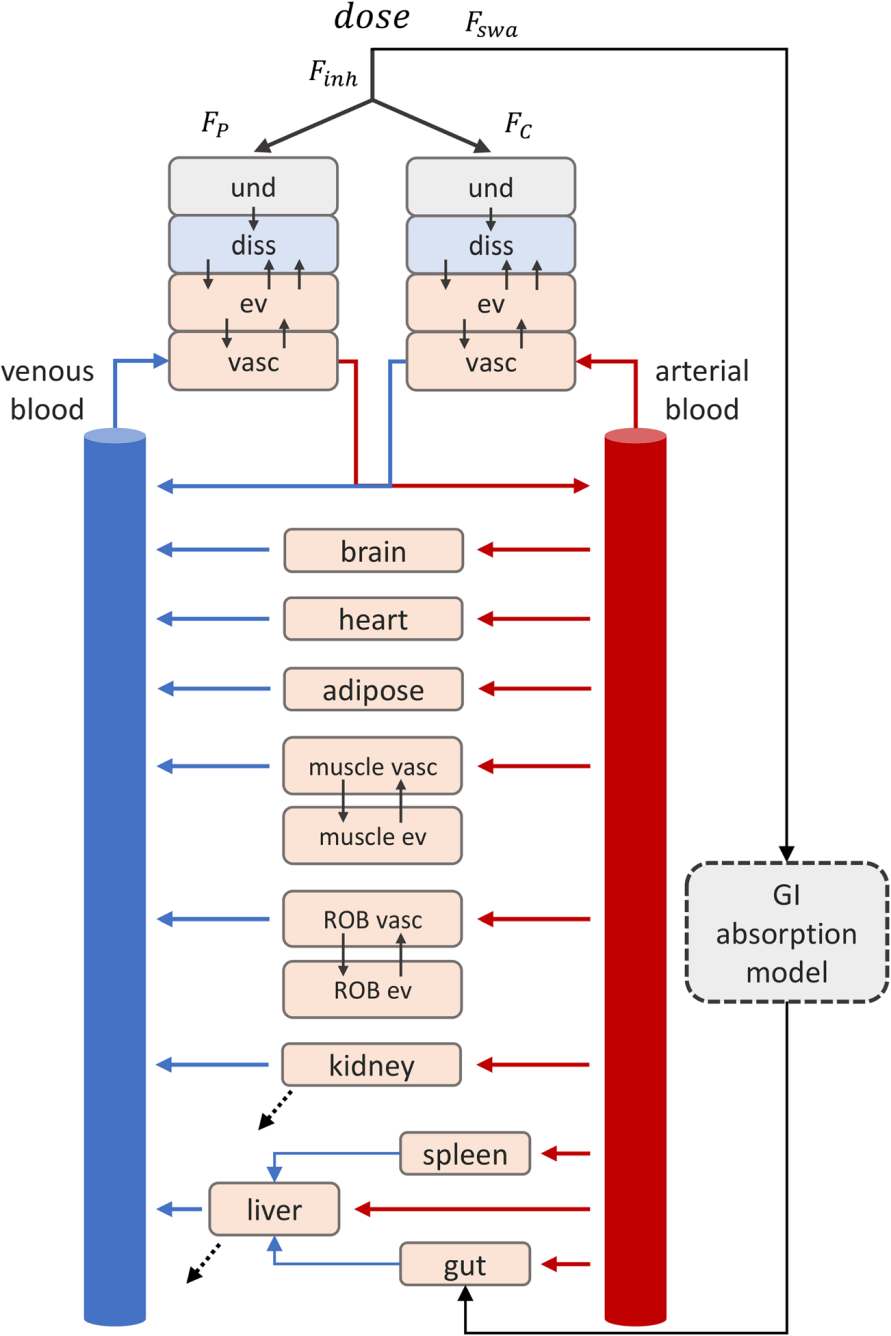
Physiologically based model structure. The model is composed of three parts: pulmonary absorption model, intestinal absorption model and systemic PBPK. Red and blue arrows represent arterial and venous blood flows, respectively. Black dotted arrows represent the clearance processes

The considered physiologically based model was originally presented in Grandoni *et al.* (5) and was inspired by the work of Boger *et al.* (19). The model is composed of three parts describing the pulmonary absorption, the intestinal absorption and the systemic disposition. The pulmonary absorption model was built to take into account the principal PK processes occurring when a drug is inhaled: deposition, mucociliary clearance, dissolution, absorption in lung tissue and in blood circulation (20). In the model, lungs were divided into two parts, the central and the peripheral regions. The central region corresponds approximatively to the tracheobronchial region, while the peripheral region to the alveolar region. Both the regions were further divided in four compartments: the undissolved drug, the dissolved drug, the extravascular and vascular lung tissue. The central region was considered perfused by the systemic circulation, while the peripheral region by the pulmonary circulation. The model structure is shown in Fig. 1.

Drugs were considered intra-tracheally administered to rats. Only a fraction of the drug amount administered to the animal actually reaches lungs (*F*_inh_), whereas the rest it is supposed to be deposited in the oropharyngeal region and gets swallowed (*F*_swa_). Of the fraction delivered to lungs, a part is deposed in the central region (*F*_*C*_) and a part reaches the peripheral region (*F*_*P*_). *F*_*C*_ was calculated from the mass median aerodynamic diameter (MMAD) and the geometric standard deviation (GSD) of the drug particles with the software MPPD2.11 (21,22), as explained in the supplementary materials, section 2. Once deposited, in both central and peripheral regions, the drug dissolves in the physiological fluids and then is supposed to be passively absorbed in the lung tissues. Here, the drug can diffuse to the vascular compartment or back to the dissolved drug compartment. A monodirectional transport from the tissue to the dissolved drug compartment was included to account for the possible action of efflux transporters, such as the P-glycoproteins. The mucociliary clearance mechanism has been considered acting only on the undissolved drug compartment of the central region, since this mechanism should be negligible in the alveoli (20).

To describe the systemic drug disposition and the intestinal absorption, the pulmonary absorption model was coupled with the whole body PBPK model presented in Grandoni *et al.* (23), as shown in Fig. 1. All the model equations are reported in the supplementary materials, section 1. Physiological lung-related parameters are reported in **Table** **SII**. The ranges of variability of physiological parameters are reported in Table I. All the remaining physiological parameter values (e.g. organ volumes and blood flows) of the whole body PBPK model are reported in the supplementary materials, section 4.

**Table I:** Physiological and Drug-Related Parameters Used for Both Inter- and Intra-compound GSA

Parameters	Reference values	Min value	Max value	References
*F*_inh_: inhaled fraction	0.9	0.9	1	Internal data
*k*_MC_: mucociliary clearance [1/h]	0.55	0.46	0.69	(34)
*w*: rat weight [kg]	0.25	0.26	0.35	Internal data
*α*: correction factor for the alveolar permeability^a^	15.6	9.2	103.57	
*GFR*: glomerular filtration rate [mL/min]	1.62	1.13 (− 30%)	2.11 (+ 30%)	(35)
*ρ*: drug true density [mg/mL]	1	0.5	1.5	(36)

^a^Permeability values were calculated from bronchial and alveolar cell layer thickness taken from (37–43)

### Inter-compound and Intra-compound GSA

**Fig. 2:**
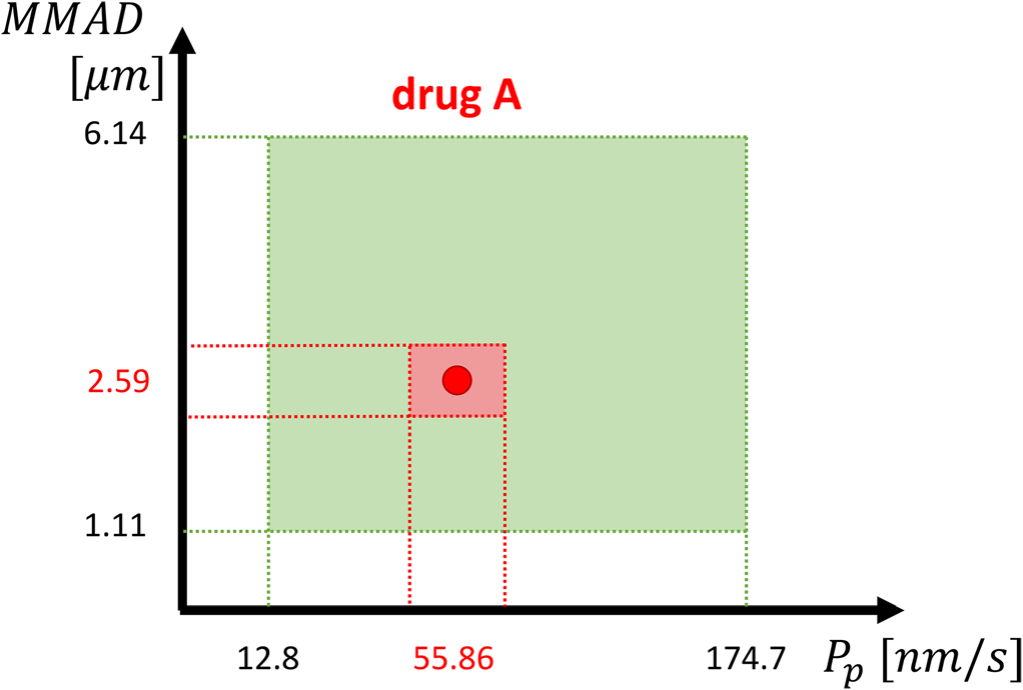
Difference in the parameter space used for *inter-compound* and *intra-compound* GSA. *Inter-compound* GSA (green area) considers the “between-drug” variability, while *intra-compound* GSA (red area) considers the uncertainty associated to the parameters in a specific compound. As shown in the figure, generally variability is wider than uncertainty

*Inter-compound* and *intra-compound* are two ways of performing GSA that differ in the aim and, thus, in the considered drug-specific parameter variability range. Figure 2 didactically shows the difference.

We performed the *inter-compound* GSA on the physiological pulmonary absorption model decoupled from the distribution PBPK and the intestinal absorption models. This was done to characterize the absorption process and to simplify the model and the results understanding. To decouple the model, blood inflow and outflow values of both peripheral and central lung vascular compartments were set equal to zero. Moreover, the fluxes due to passive permeability from the vascular to the extravascular compartments were set equal to zero too, in both the lung regions. Thus, lung vascular compartments behave like traps (integrators). The outputs considered in this analysis are the fraction absorbed (*f*_a_) and the AUC and MRT of the drug concentration in the whole lung. Whole lung concentration was obtained as the sum of the solid and dissolved amounts in the epithelial lining fluids with the amounts in the tracheobronchial and alveolar extravascular compartments, divided by the total volume. *f*_a_ was obtained as *f*_a_ = 1 − *f*_*CL*_, where *f*_*CL*_ is the fraction of the drug eliminated by the mucociliary clearance at the steady state.

In the *inter-compound* GSA, we considered each of the drug-related parameters varying in a range given by the minimum and the maximum values observed in a set of nine compounds belonging to the *Chiesi Farmaceutici* portfolio (Table II). The parameter distributions were considered uniform in these ranges. The PBPK model performances on these nine compounds are shown in the supplementary materials, section 5.

**Table II:** Drug-Related Parameters Used for Inter-compound GSA

Parameters^a^	Min value	Max value
*mw*: molecular weight [g/mol]	334.4	769.2
*MMAD*: mass median aerodynamic diameter [10^−4^ cm]^b^	1.11	6.14
*GSD*: geometric standard deviation of the aerodynamic diameters [10^−4^ cm]^b^	0.84	3.432
*D*_0, *inh*_: dose number	0.045 (1)	1 (160.89)
*f*_*u*, *t*_: fraction unbound in lung tissue	0.001	0.26
*f*_*u*, *elf*_: fraction unbound in epithelial lung fluid	0.1	1
*P*_*p*_ *Calu*3: fitted passive permeability [nm/s] ^c^	12.85	174.7
*P*_*a*_ *Calu*3: fitted active permeability [nm/s] ^c^	4e-6	60,600

^a^All the data were internally available

^b^Used to compute the deposition fraction as explained in the supplementary materials, section 2

^c^Permeability values were calculated as explained in the supplementary materials, section 3

To have more homogeneous behaviour, *inter-compound* GSA was performed separately for highly and poorly soluble compounds. The criterion used to classify compounds in the two groups was inspired to the one adopted for orally administered compounds (24–26). A dose number for inhaled compounds was defined as in Eq. (4).

4 $$ {D}_{0,\mathrm{inh}}=\frac{\mathrm{dose}/{V}_{\mathrm{elf}}}{C_{\mathrm{S},\mathrm{p}{\mathrm{H}}_{\mathrm{elf}}}} $$

Drug dose was fixed to 10 μg (i.e. the typical selected dose in the considered experiments), *V*_elf_ is the lung epithelial lining fluid (ELF) volume (sum of the central and peripheral ELF volumes) and ${C}_{\mathrm{S},\mathrm{p}{\mathrm{H}}_{\mathrm{elf}}}$ is the drug solubility measured in simulated lung fluid at pH 6.9 (27,28). A compound was classified as highly soluble if *D*_0, inh_ < 1 or poorly soluble if *D*_0, inh_ ≥ 1. During GSA, we first extracted the values of *D*_0, inh_ and then we computed the ${C}_{\mathrm{S},\mathrm{p}{\mathrm{H}}_{\mathrm{elf}}}$ using Eq. (4) (6).

The *intra-compound* GSA was performed on the whole body PBPK model for three representative compounds, characterized by different properties. The considered outputs are the drug whole lung and plasma concentration AUC and MRT. Here, whole lung concentration was calculated as the sum of the drug amount in all the pulmonary absorption model compartments, divided by the lung total volume. All the drug-specific model parameters were considered uniformly distributed between the ranges reported in Table III, except for the dose that was considered normally distributed with a CV equal to 15% (29). When no experimental data supporting the uncertainty range definition were available, ranges reflecting the perceived parameter uncertainties were used.

**Table III:** Drug A, B and C Parameter Ranges for *Intra-compound* GSA

Parameters	Drug A^a^	Drug B^a^	Drug C^a^
*dose*: drug dose [μg] ^b^	10	15	23
*logP*^c^	3.87 (± 30%)	1.99 (± 30%)	5.4 (± 30%)
*pKa*	8.7 (± 0.1)	9.81 (± 0.1)	8.5 (± 0.1)
*P*_*p*_ *Calu*3: fitted passive permeability [nm/s]	55.86 (± 70%)	16.06 (± 70%)	20.54 (± 70%)
*P*_*a*_ *Calu*3: fitted active permeability [nm/s]	4 ∙ 10^−6^ (± 70%)	68.82 (± 70%)	598.74 (± 70%)
*CACO*2_*AB*_: gut wall permeability [*nm*/*s*]	4.7 (± 70%)	49.2 (± 70%)	0.3 (± 70%)
*BP*: blood to plasma ratio	0.8 (± 10%)	1.6 (±10%)	1 (± 10%)
*E*_*r*_: Extraction ratio, derived from IV Non-Compartmental Analysis	0.8 (± 30%)^e^	0.95 (±30%) ^e^	0.95 (± 30%)^e^
*S*: drug solubility [ng/ml]	696 (± 30%)	360,000 (± 30%)	14,300 (± 30%)
*MMAD*: mass median aerodynamic diameter [μm]^d^	2.59 (± 30%)	3.2 (± 30%)	1.71 (± 30%)
*GSD*: geometric standard deviation [μm]^d^	2.1 (±30%)	1.67 (± 30%)	2.33 (± 30%)
*F*_inh_: fraction inhaled	0.9–1	0.9–1	0.9–1
*f*_u, elf_: fraction unbound in epithelial lung fluid	0.16 (± 30%)	1 (± 30%)^e^	0.1 (± 30%)
*f*_u, t_: fraction unbound in lung tissue	0.0015 (± 30%)	0.26 (± 30%)	0.001 (± 30%)
*f*_up_: fraction unbound plasma	0.032 (± 30%)	0.82 (± 30%)^e^	0.0015 (± 30%)

^a^Minimum or maximum range limit (difference with respect to the baseline value, in percentage)

^b^The dose was considered normally distributed with a CV equal to 15% (29)

^c^The ranges were calculated as ± 30% of the natural value

^d^Ranges were set equal to − 30% the minimum and + 30% the maximum of multiple measurements

^e^The upper limit was set equal to 1

To account for the population variability of rat weight, all the volumes and blood flows were multiplied for (*w*_subj_/*w*_mean_) and (*w*_subj_/*w*_mean_)^0.75^, respectively, where *w*_subj_ is the extracted rat weight and *w*_mean_ is the mean rat weight equal to 250 g, as used in (23).

All the analyses were performed by using MATLAB R2019a (30) on a 64-bit computer configured with Intel® Core™ i7-7700 @ 3.60 GHz x8 processors, running Ubuntu 16.04 LTS. The differential equations were solved by using the ‘ode15s’ MATLAB solver, for a time span ranging from 0 to 400 h. An *ad hoc* MATALB code to implement variance-based GSA was developed. To perform the GSA in both *inter-* and *intra-compound* cases, we used 20,000 samples (*n*) extracted from a unit hypercube. Then, samples were brought back to each parameter distribution by using simple linear transformations for the uniformly distributed parameters and the inverse cumulative density function for the other parameters. *n* number of samples corresponds to *n*(*k* + 2) number of model evaluation needed to compute the sensitivity indices, with *k* the number of parameters (17). Uncertainty on the calculation of the sensitivity indices was estimated by using 10,000 bootstrap samples (31).

## RESULTS

### Inter-compound GSA

**Fig. 3:**
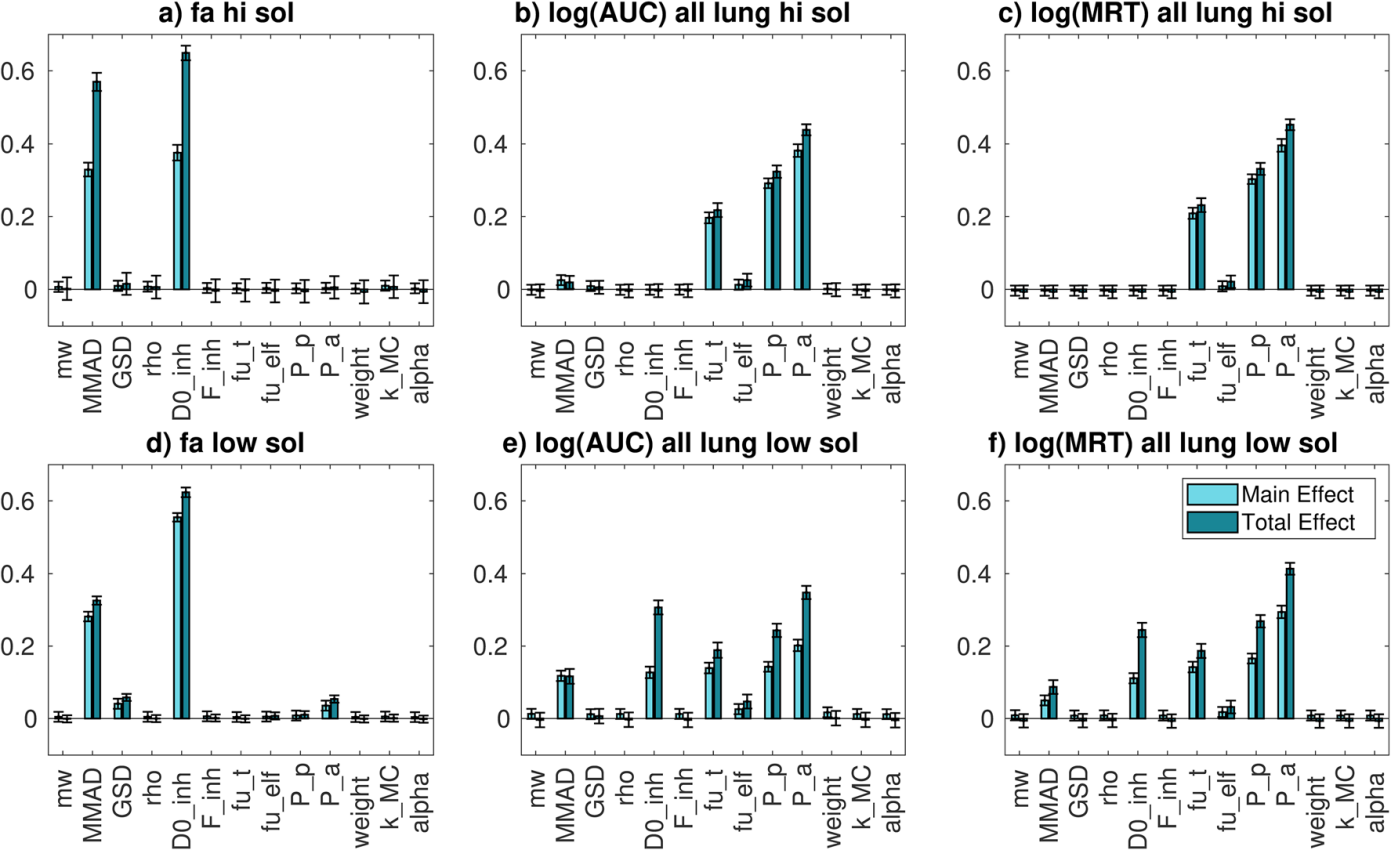
*Inter-compound* GSA results for the output of interests: **a** fraction absorbed for highly soluble compounds; **b** logarithm of whole lung AUC for highly soluble compounds; **c** logarithm of whole lung MRT for highly soluble compounds; **d** fraction absorbed for poorly soluble compounds; **e** logarithm of whole lung AUC for poorly soluble compounds; **f** logarithm of whole lung MRT for poorly soluble compounds. Concerning the parameters: *mw* is the compound molecular weight; *MMAD* is the mass median aerodynamic diameter; *GSD* is the geometric standard deviation; *rho* the drug true density; *D0_inh* is the dose number; *F_inh* the inhaled fraction; *fu_t* is the fraction unbound in lung tissue; *fu_elf* is the fraction unbound in the epithelial lining fluid; *P_p* is the passive permeability; *P_a* is the active permeability; *weight* is the rat body weigh; *k_MC* is the mucociliary clearance; *alpha* is the scaling factor for the peripheral lung passive permeability

Here, the results of the *inter-compound* GSA on the pulmonary absorption model decoupled from the whole-body PBPK, for both highly and poorly soluble compounds, are reported. References to figures reported in the supplementary materials are indicated with an “S”, followed by an Arabic number. In **Fig.** **S7**, the distributions of the selected model outputs are shown. In Fig. 3 the sensitivity indices for *f*_a_ and for the logarithms of drug concentration AUC and MRT in lungs are shown. We choose the log scale for the AUC and MRT to avoid problems in the computation of the variance-based sensitivity index estimation due to the skewness of AUC and MRT distributions in natural scale (32).

For highly soluble compounds, the parameters that mostly explain the *f*_a_ variability are *D*_0, inh_ and *MMAD*. Concerning *D*_0, inh_, it is probably important because it controls the solubility, and so the dissolution rate. The higher the dissolution rate is, the faster the drug is removed from the solid compartment in the central region. That is in fact the region in which the drug could be eliminated via mucociliary clearance. However, the *f*_a_ variability is quite low, as shown in **Figure** **S7**. So, *D*_0, inh_ and *MMAD* are the most important parameters, but the variation of the model output is in any case limited.

For poorly soluble compounds, both *MMAD* and *D*_0, inh_, even if with a minor contribution of *GSD* and the active permeability (*P*_a_), still impact the *f*_a_ variability. Concerning *D*_0, inh_, the reasons of its importance are probably the same of highly soluble compounds. *MMAD* could be important in determining *f*_a_ variability mainly for two reasons: first of all, the *MMAD* value could impact the dissolution rate when the solubility is low; second, it determines *F*_*C*_ and, mainly for poorly soluble compounds, *f*_a_ could be sensitive to the repartition between central and peripheral regions. In fact, in peripheral region the mucociliary clearance does not occur. The main difference with respect to the high solubility case is that *f*_a_ variability is quite high. Thus, *MMAD* and *D*_0, inh_ are responsible for a great variation of the model output.

Concerning lung AUC, the parameters that mostly explain its variability for highly soluble compounds are *f*_u, t_ (fraction unbound in the lung tissue), *P*_p_ (passive permeability) and *P*_a_. This probably happens because *f*_u, t_ and the permeabilities are parameters that determine the drug retention into the lungs and thus they control the AUC. For poorly soluble compounds, in addition to parameters that control the drug retention into the lungs, *D*_0, inh_ and *MMAD* are also important. This happens because, as explained before, they could impact *f*_a_.

Concerning lung MRT, the parameters that mostly explain the variability for highly soluble compounds are still *f*_u, t_ and both passive and active permeabilities. The reasons of their importance are probably similar to the one for the AUC: these are the parameters responsible to the drug retention in lungs. For poorly soluble compounds, the most important parameters are the same of highly soluble compounds, with the addition of *D*_0, inh_ and *MMAD*.

### Intra-compound GSA

Here the results of the *intra-compound* GSA for three representative compounds belonging to the Chiesi Farmaceutici portfolio, namely A, B and C, are reported. With respect to all the other compounds, compound A is characterized by a lower solubility, a higher permeability and a low *f*_u, t_. Compound B has a higher solubility, a lower permeability and a higher *f*_*u*, *t*_. Finally, compound C has an intermediate solubility and permeability and a low *f*_u, t_. The parameter values and associated uncertainty or variability are reported in Table III.

#### Compound A

**Fig. 4:**
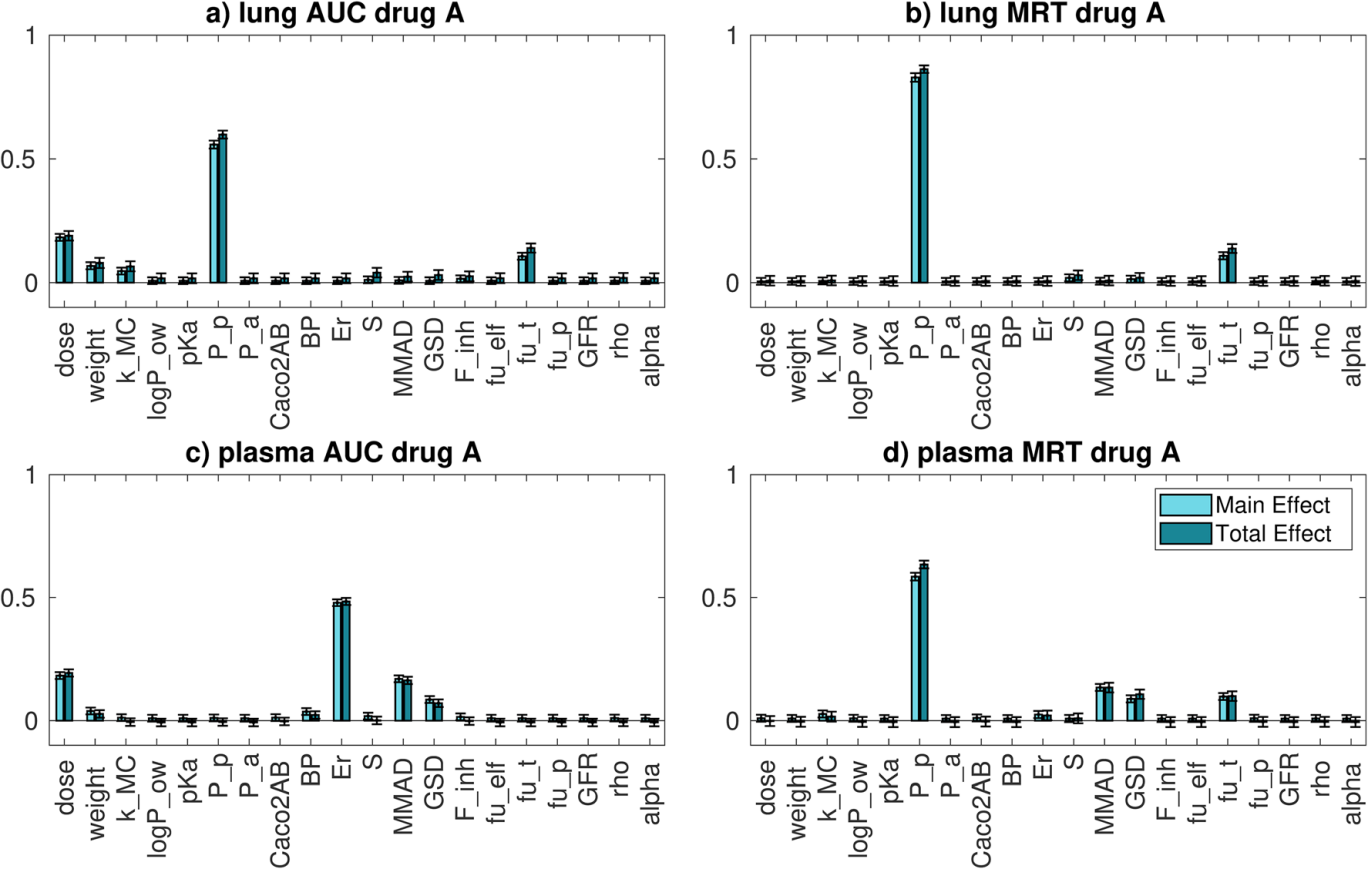
*Intra-compound* GSA results for **a** whole lung AUC, **b** whole lung MRT, **c** plasma AUC and **d** plasma MRT for compound A. Concerning the parameters: *dose* is the drug dose; *weight* is the rat body weight; *k_MC* is the mucociliary clearance; *logP_ow* is the logarithm of the octanol-water partition coefficient; *pKa* is the drug pKa; *P_p* is the passive permeability; *P_a* is the active permeability; *Caco2AB* is the permeability calculated using the CACO2 cell layer; *BP* is the blood to plasma ratio; *Er* is the extraction ratio; *S* is the drug solubility; *MMAD* is the mass median aerodynamic diameter; *GSD* is the geometric standard deviation; *F_inh* the inhaled fraction; *fu_elf* is the fraction unbound in the epithelial lining fluid; *fu_t* is the fraction unbound in lung tissue; *fu_p* is the fraction unbound in plasma; *GFR* is the glomerular filtration rate; *rho* is the drug true density; *alpha* is the scaling factor for the peripheral lung passive permeability

The distribution of drug plasma and whole lung AUC and MRT and the sensitivity indices relative to compound A are reported in **Figure** **S8** and Fig. 4, respectively.

Whole lung AUC variance is mainly explained by the *P*_p_ uncertainty, together with a minor contribution of the dose, *f*_u, t_, rat weight and *k*_*MC*_ (mucociliary clearance) variabilities. Even if the drug has a low solubility, it seems that the highest impact on the AUC is attributed to the passive permeability. This probably happens because compound A has very low fraction unbound in tissue; thus, even if the permeability of the free compound is relatively high, the overall drug flux across the cell membrane results to be the rate limiting step. In addition to that, it should be noted that the uncertainty associated to the passive permeability is higher with respect to those associated to other parameters. The passive permeability is still the most important parameter when whole lung MRT is considered. The reasons seem to be similar to those discussed for the AUC. Plasma AUC variability is mainly explained by the extraction ratio and, to a minor extent, by the dose, *MMAD* and *GSD* variabilities. These results highlight that the elimination process plays a major role in determining the plasma AUC variability. Unless nonlinear processes are involved, the importance of the dose and the extraction ratio on the plasma AUC is completely expected from a PK point of view. Concerning the plasma MRT, the most important parameter is the passive permeability. This probably happens because the drug is slowly absorbed from the lungs into the systemic circulation.

#### Compound B

**Fig. 5:**
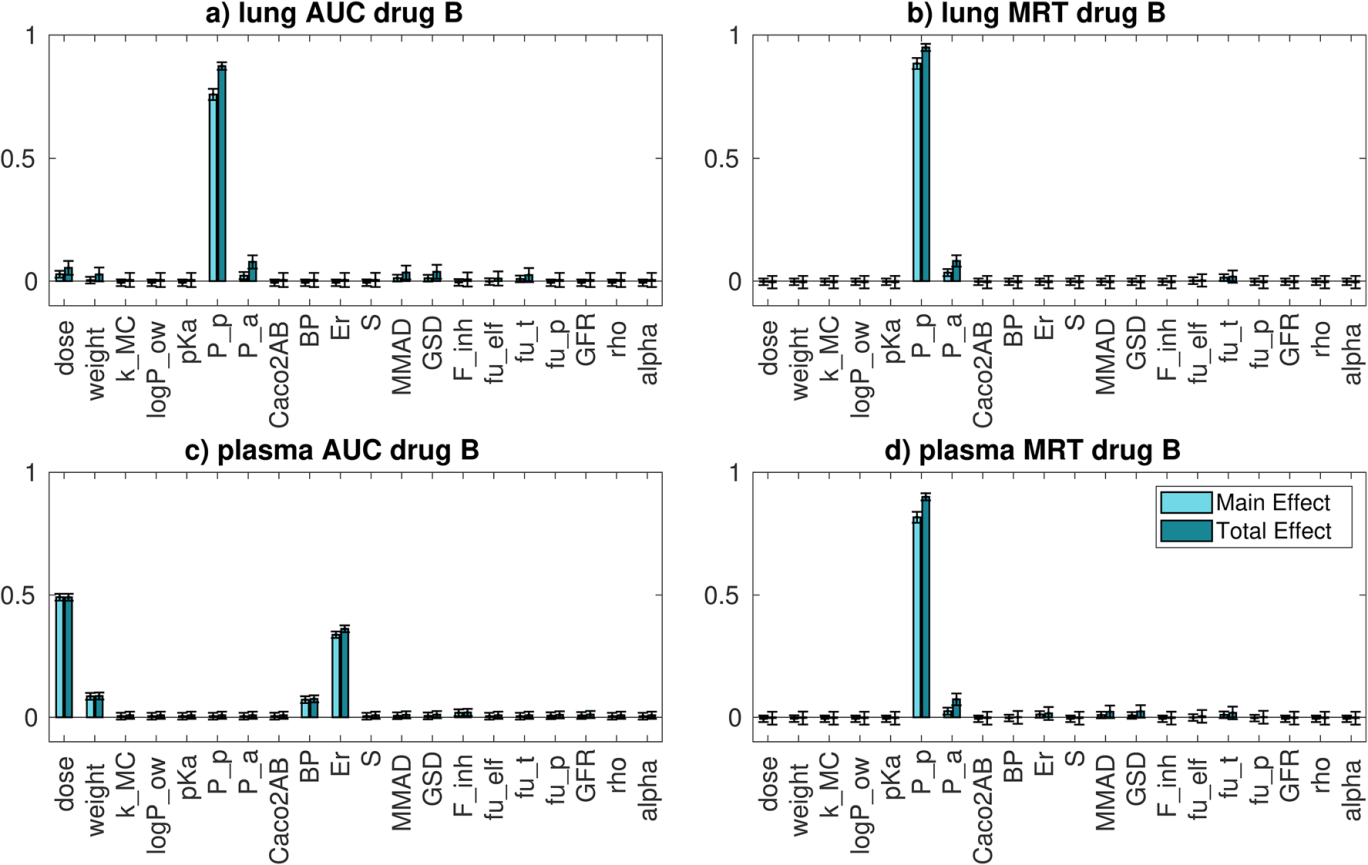
*Intra-compound* GSA results for **a** whole lung AUC, **b** whole lung MRT, **c** plasma AUC and **d** plasma MRT for compound B. For the parameters’ acronyms, please refer to Fig. 4 label

The distribution of drug plasma and whole lung AUC and MRT and the sensitivity indices for relative to compound B are reported in **Figure** **S9** and Fig. 5, respectively.

The parameter that mostly explains compound B whole lung AUC and MRT variation is *P*_p_. This probably happens because compound B has a lower permeability and higher solubility with respect to the other compounds of interest; thus, the absorption rate is probably permeability limited. Moreover, as explained for compound A, with respect to all the other parameters, the permeability has associated a greater uncertainty; thus, it is more likely that they have a relevant impact in explaining the AUC variation.

Concerning the plasma AUC, the most important parameters are the dose and the extraction ratio, followed by rat weight and the blood to plasma ratio (BP). As for compound A, the elimination process is more important than the distribution or absorption processes in determining the AUC variation. The most important parameter in explaining the MRT variance is the passive permeability. As for compound A, this probably happens because the drug is slowly absorbed from the lungs into the systemic circulation.

#### Compound C

**Fig. 6:**
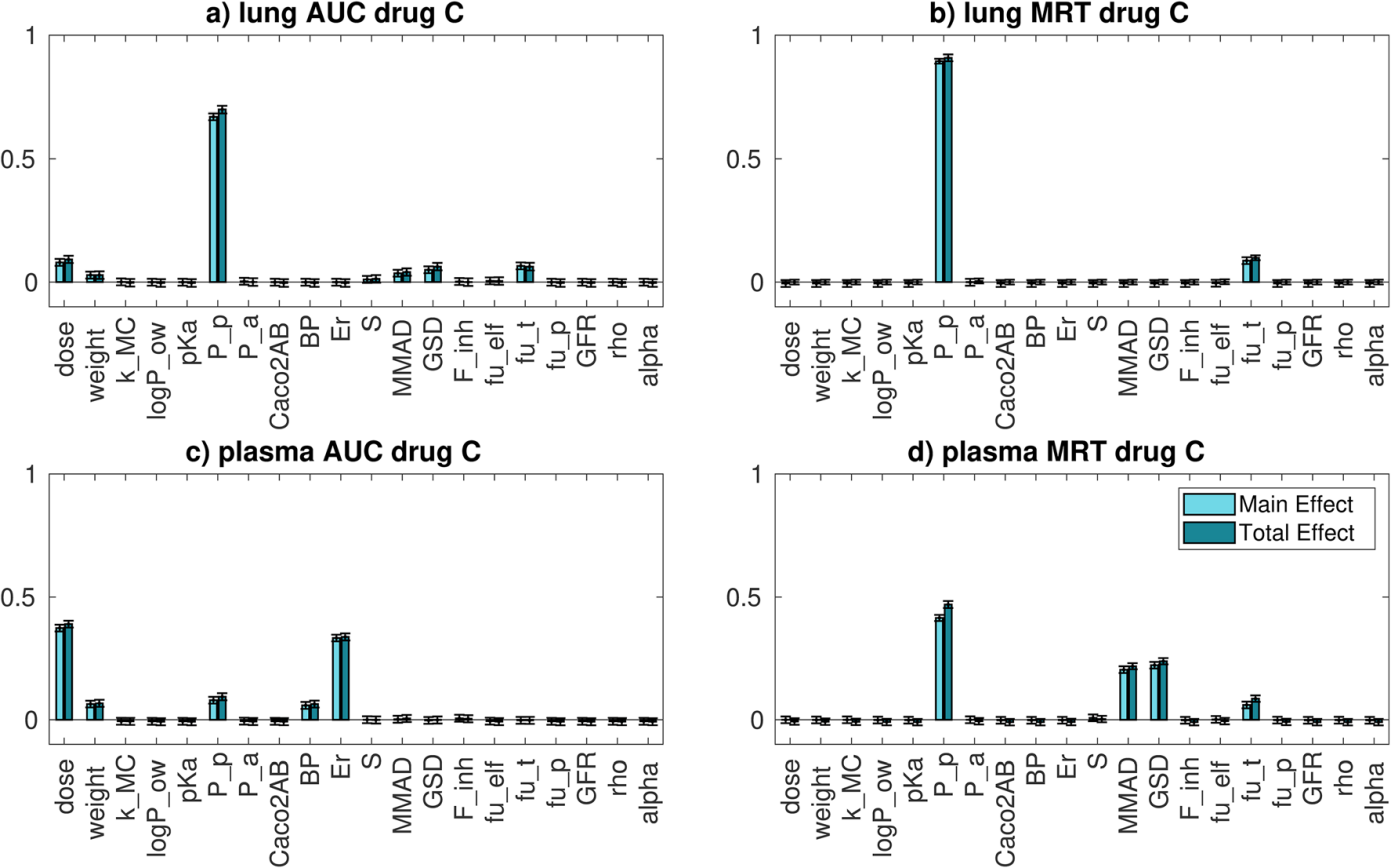
*Intra-compound* GSA results for **a** whole lung AUC, **b** whole lung MRT, **c** plasma AUC and **d** plasma MRT for compound C. For the parameters’ acronyms, please refer to Fig. 4 label

The distribution of plasma and whole lung AUC and MRT and the sensitivity indices relative to compound C are reported in **Figure** **S10** and Fig. 6, respectively.

For both whole lung AUC and MRT, the parameter that with its variation mainly explains their variability is *P*_p_. The reasons are probably similar to those reported for compounds A and B. Moreover, compound C has a higher solubility, but lower permeability than compound A and, together with a low fraction unbound, this could explain the slightly higher importance of the passive permeability with respect to compound A.

Concerning the plasma AUC, the parameters that mainly explain its variation are the dose and the extraction ratio, followed by the passive permeability, rat weight and BP. These results highlight that probably, for this drug, the elimination process has a greater role in determining the AUC variability with respect to the distribution or absorption. Concerning the plasma MRT, the situation resembles the one of compound A.

## DISCUSSION

In this manuscript, we showed how GSA techniques were used to assess model behaviours and support the development of a mechanistic model describing pulmonary absorption for inhaled compounds. We identified two ways of performing GSA that differ in the aims and, thus, in the considered parameter variability: *inter-compounds* and *intra-compound*. Both the approaches helped in understanding different model aspects.

The *inter-compound* GSA was performed for the absorption model decoupled from the distribution PBPK. This analysis can be performed in more “homogeneous” sub-spaces of the whole parameter space, as we have done distinguishing highly soluble from poorly soluble compounds. Looking at the model output distributions gives the possibility of assessing the extent of the *inter-compound* variability of the metrics of interest. Then, GSA helps in understanding what the parameters are that mostly determine the observed variation of the output predictions between different compounds. For example, from our analysis it was possible to understand that the between-compound differences in the lung AUC for the low solubility compounds are mainly driven by the variation of both solubility- and permeability-related parameters and that strong interaction effects are present. In particular, the latter means that the effect of solubility and permeability on the lung AUC is not additive. This information may be particularly useful if the *inter-compound* GSA results are used to inform the optimization of the compounds’ physicochemical properties. As a consequence of performing *inter-compound* GSA, we gained insights on the model behaviour and, consequently, we increased the knowledge of the model. For these characteristics, *inter-compound* GSA is particularly useful for the error detection. In fact, in case of discrepancies between the expected and the actual model behaviours, GSA gives useful information that helps in identifying the reasons and, thus, possible errors in the model assumptions or implementation. When the *inter-compound* GSA was applied for the first time to our in-house PBPK model (that was still in the development phase), we immediately noticed that the model did not behave as expected. Guided by the GSA results, we found and corrected the implementation error. The implementation of complex PBPK models often consists of thousands of lines of code and hundreds of parameters (this is particularly true for commercial PBPK software). A ‘little bug’ in the code may have a huge impact on the model behaviour. For its ability to assess the model behaviour, to help in the error detection and, as reported by Iooss and Saltelli, for the general GSA capability of helping in identifying sensitive assumptions (33), the *inter-compound* GSA is particularly useful during the process of model development. Theoretically, if the model structure and the physiological and *inter-compound* parameter variabilities are correctly identified and fixed, this analysis can be performed just once (e.g. when the model is firstly presented or at the PBPK platform release).

The *intra-compound* GSA was instead performed for three representative compounds on the whole body PBPK model. The parameter variation was defined to represent the uncertainties associated to their values for a specific compound. With this analysis it is possible to know how much the model output variation is apportioned to the uncertainty of the parameters. When doing this analysis, it is useful to look at the output distribution, to determine if it is narrow enough to be considered acceptable. If not, GSA helps in selecting which parameters should be known with less degree of uncertainty in order to give a more accurate prediction. For example, we believe that the uncertainty associated to compound A lung MRT is too high. So, if one is interested in using this model for lung MRT predictions (e.g. for different dosages or species), from the GSA results we know that a better characterization of the passive permeability is needed in order to reduce uncertainties of the considered metrics. This situation probably does not happen for compound A lung AUC, given that we believe that the uncertainty associated with the prediction of this metric can be considered acceptable. Differently from *inter-compound* GSA, *intra-compound* GSA should be performed each time the model (or the PBPK platform) is used for a specific drug.

One challenge that we have faced in performing GSA was that, in certain situations, determining the uncertainty or variability ranges was not an easy task, in particular for the *intra*-*compound* GSA. In fact, due to the lack of available data, it was sometimes difficult to appropriately quantify the parameters’ degree of uncertainty. In these cases, expert opinion can be used to fill this gap.

In our work, we presented the applications of *inter-compound* and *intra-compound* GSA in a preclinical setting involving animal models. However, the use of these analyses is not limited to this context. The results of the *inter-compound* GSA highlight how a simultaneous variation of the compounds’ physicochemical properties impacts the model outputs. This information could be particularly precious in guiding compound optimization in early discovery phases. Moreover, similarly to what was shown here, the *inter-compound* GSA can be used in clinics to understand how the model behaves varying together both drug- and subject-specific parameters. Moreover, a comparison of the clinical *inter-compound* GSA results with the ones from the preclinical setting could give insights on how physiological changes across the species impact the model results. *Intra-compound* GSA can be useful outside of the preclinical setting as well. For its characteristics, this analysis is particularly suitable when the model is used to predict unseen scenarios, such as for the first in human translation and for predicting the PK in different populations (e.g. paediatrics).

## CONCLUSION

In this work, we showed how GSA can be used within PBPK modelling and simulation. Performing GSA during both the model development and routine use increases the knowledge of the model; it helps in finding errors and in identifying the parameters that must be known with higher confidence, if one is interested in reducing the model prediction uncertainties. GSA is a crucial instrument for the quality assessment of model-based inference; for this reason, we suggest its use during both PBPK model development and use.

## Electronic supplementary material

ESM 1 (PDF 694 kb)
