# Supplementary material for: Inter-compound and Intra-compound Global Sensitivity Analysis of a Physiological Model for Pulmonary Absorption of Inhaled Compounds
Source: AAPS J. 2020 Aug 30;22(5):116. doi: 10.1208/s12248-020-00499-0 (PMC7456635; doi:10.1208/s12248-020-00499-0)
Supplement: Supplementary file 1 — (PDF 694 kb) [file 12248_2020_499_MOESM1_ESM.pdf]

Inter-compound and *intra-compound* global sensitivity analysis of a physiological model for pulmonary absorption of inhaled compounds

Supplementary Material

The AAPS Journal

Nicola Melillo[1], Silvia Grandoni[1], Nicola Cesari[2], Giandomenico Brogin[2], Paola Puccini[2], Paolo Magni[1,*]

[1]Laboratory of Bioinformatics, Mathematical Modelling and Synthetic Biology, Department of Electrical, Computer and Biomedical Engineering, Università degli Studi di Pavia, Pavia, Italy

[2]Pharmacokinetic, Biochemistry and Metabolism Department, Chiesi Farmaceutici S.p.A., Parma, Italy

***Email**: paolo.magni@unipv.it

1 PBPK model equations

The pulmonary absorption model was previously published in (1), while the whole body PBPK model in (2).

1.1 Pulmonary absorption model

The pulmonary absorption model equations are shown in equation system (S1).

$$
\begin{aligned}
\frac{da_{u,C}}{dt} &= -(k_{d,C} + k_{MC})\, a_{u,C} \\
\frac{da_{d,C}}{dt} &= k_{d,C}\, a_{u,C} - P_p\, S_C\, \frac{a_{d,C}\, f_{u,elf}}{V_{elf,C}} + (P_p + P_a)\, S_C\, \frac{a_{ev,C}\, f_{u,t}}{V_{ev,C}} \\
\frac{da_{ev,C}}{dt} &= P_p\, S_C\, \frac{a_{d,C}\, f_{u,elf}}{V_{elf,C}} + P_p\, S_C\, \frac{a_{v,C}\, f_{u,b}}{V_{v,C}} - (2P_p + P_a)\, S_C\, \frac{a_{ev,C}\, f_{u,t}}{V_{ev,C}} \\
\frac{da_{v,C}}{dt} &= Q_C \left(c_{art} - \frac{a_{v,C}}{V_{v,C}} \right) + P_p\, S_C\, \frac{a_{ev,C}\, f_{u,t}}{V_{ev,C}} - P_p\, S_C\, \frac{a_{v,C}\, f_{u,b}}{V_{v,C}} \\
\frac{da_{u,P}}{dt} &= -k_{d,P}\, a_{u,P} \\
\frac{da_{d,P}}{dt} &= k_{d,P}\, a_{u,P} - \alpha P_p\, S_P\, \frac{a_{d,P}\, f_{u,elf}}{V_{elf,P}} + (\alpha P_p + P_a)\, S_P\, \frac{a_{ev,P}\, f_{u,t}}{V_{ev,P}} \\
\frac{da_{ev,P}}{dt} &= \alpha\, P_p\, S_P\, \frac{a_{d,P}\, f_{u,elf}}{V_{elf,P}} + \alpha\, P_p\, S_P\, \frac{a_{v,P}\, f_{u,b}}{V_{v,P}} - (2\alpha P_p + P_a)\, S_P\, \frac{a_{ev,P}\, f_{u,t}}{V_{ev,P}} \\
\frac{da_{v,P}}{dt} &= Q_P \left(c_{ven} - \frac{a_{v,P}}{V_{v,P}} \right) + \alpha P_p\, S_P\, \frac{a_{ev,P}\, f_{u,t}}{V_{ev,P}} - \alpha P_p\, S_P\, \frac{a_{v,P}\, f_{u,b}}{V_{v,P}}
\end{aligned}
\tag{S1}
$$

Subscripts C and P stand for central and peripheral lung regions, respectively; a_u, a_d, a_{ev} and a_v are the drug amounts in undissolved, dissolved, extravascular and vascular compartments, respectively; c_{ven} and c_{art} are the drug concentrations in venous and arterial compartments; $f_{u,elf}$, $f_{u,t}$ and $f_{u,b}$ are the drug fraction unbound in the epithelial lining fluids (ELF), lung tissues and blood; V_{elf}, V_{ev} and V_v are the volumes of the ELF and the extravascular and vascular compartments, respectively; Q_P and Q_C are the blood flows directed to the peripheral and central lung regions; P_p and P_A are the passive and active permeabilities, calculated from experiments with Calu3 cells (see section 3), which have characteristics similar to the tracheobronchial region (3–5); S_P and S_C are the surfaces of peripheral and central regions; k_{MC} is the time constant relative to mucociliary elimination, supposed to happen only in central region, while k_d it is the dissolution time constant, modelled with the Noyes-Whitney model (6), as reported in the following equation.

$$
k_d = \frac{3D}{\rho\, r\, h} \left(C_S - \frac{a_d\, f_{u,elf}}{V_{elf}} \right) \tag{S2}
$$

D is the drug diffusion coefficient, ρ is the drug density, r is the radius of the particle size of the formulation, h is the diffusional layer thickness and C_S is the drug solubility at lung epithelial lining fluids pH. r is computed from the mass median aerodynamic diameter (MMAD) by

$$r = r_a / \sqrt{\frac{\rho/\rho_0}{\chi}} \quad \text{(S3)}$$

where r_a is the aerodynamic radius (assumed equal to MMAD/2), ρ_0 is the unit density (1 g/cm^3) and χ is the shape factor, that was set equal to 1 (thus, assuming that the particle shape is spherical) (7).

D is calculated with the Stokes-Einstein equation, as reported in the equation below.

$$D = \frac{k_B\, T}{6\pi\, \eta_{slf}\, R_s} \quad \text{(S4)}$$

k_B is the Boltzmann constant, T is the absolute body temperature in Kelvin, η_{slf} is the simulated lung fluid (SLF) viscosity and R_s is the hydrodynamic radius, showed below (8).

$$R_s = \sqrt[3]{\frac{3\, mw}{4\pi\, N_A\, \rho}} \quad \text{(S5)}$$

mw is the drug molecular weight and N_A is the Avogadro's number. The diffusional layer thickness h was derived following the approximation of Hintz and Johnson (9):

$$h = r, \qquad if \;\; r < 30\ \mu m$$
$$h = 30\mu m, \qquad otherwhise$$

In equation system (S1), α is a scalar constant used to model the higher passive permeability in peripheral region, with respect to the central one, due to the minor thickness of alveolar epithelium. α was calculated as follows.

$$\alpha = \frac{BT}{ALT} \quad \text{(S6)}$$

BT and ALT are the thickness of the bronchial and alveolar wall, respectively.

1.2 PBPK for drug distribution

The mass balance equation for the brain, heart, adipose tissue and spleen is reported in equation (S7) (10).

$$\frac{dC_T}{dt} = \frac{Q_T}{V_T}\left(c_{art} - \frac{c_T}{P_{T:B}}\right) \tag{S7}$$

V_T and Q_T are the tissue volume and blood flux; $P_{T:B}$ is the tissue to blood partition coefficient.

The equation for the venous compartment is reported below (11):

$$\frac{dc_{ven}}{dt} = \frac{1}{V_{ven}}\left[\sum_T \left(\frac{Q_T c_T}{P_{T:B}}\right) - Q_{CO} c_{ven}\right] \tag{S8}$$

where V_{ven} is the venous drug volume and Q_{CO} is the cardiac output. The mass balance equation for the arterial compartment is reported below (11):

$$\frac{dc_{art}}{dt} = \frac{Q_{CO}}{V_{art}}\left(\frac{a_{v,P}}{V_{v,P}} - c_{art}\right) \tag{S9}$$

where V_{art} is the arterial drug volume.

The drug distribution in the muscles and in the rest of the body compartment (that includes the bones and the skin) was described with a permeability limited model, as reported in equation X (12).

$$\begin{aligned} \frac{dc_{v,T}}{dt} &= \frac{1}{V_{v,T}}\left(Q_T c_{art} - Q_T c_{v,T} - PS \cdot c_{v,T} f_{u,b} + PS \cdot c_{ev,T}\, f_{u,T}\right) \\ \frac{dc_{ev,T}}{dt} &= \frac{1}{V_{ev,T}}\left(PS \cdot c_{v,T} f_{u,b} - PS \cdot c_{ev,T} f_{uT}\right) \end{aligned} \tag{S10}$$

$c_{v,T}$ is the drug concentration in the vascular part of the tissue T and $V_{v,T}$ is its volume; $c_{ev,T}$ and $V_{ev,T}$ are the concentration and volume of the extra vascular part of the tissue; PS is the permeability-surface product.

The liver mass balance is reported in equation (S11) (11).

$$\begin{aligned} \frac{dc_{liv}}{dt} = \frac{1}{V_{liv}}&\left[\left(Q_{liv} - Q_{gut} - Q_{spl}\right)c_{art} + Q_{gut}\frac{c_{gut}}{P_{gut:B}} + Q_{spl}\frac{c_{spl}}{P_{spl:B}}\right] \cdot (1 - E_R) \\ &- \frac{Q_{liv}}{V_{liv}} \cdot \frac{c_{liv}}{P_{liv:B}} \end{aligned} \tag{S11}$$

V_{liv} is the liver volume, c_{liv}, Q_{liv}, $P_{liv:B}$ are the liver concentration, total flux and tissue blood partition coefficient respectively. c_{gut}, Q_{gut}, $P_{gut:B}$ are those of the gut and C_{spl}, Q_{spl}, $P_{spl:B}$ are those of the spleen. E_R is the liver extraction ratio.

The mass balance equation for kidney has been formulated in its simplest form considering the passive filtration mechanism governed by the glomerular filtration rate and neglecting processes of active secretion and tubular reabsorption (13).

$$\frac{dc_{kidney}}{dt} = \frac{1}{V_{kidney}} \left[Q_{kidney} \left(c_{art} - \frac{c_{kidney}}{P_{kidney:B}} \right) - GFR\, f_{u,b}\, c_{art} \right] \quad \text{(S12)}$$

where c_{kidney} is the kidney concentration, V_{kidney} and Q_{kidney} are the kidney volume and blood flow and GFR is the glomerular filtration rate.

The mass balance equation for the gut tissue has been modelled as follows (13):

$$\frac{dc_{gut}}{dt} = \frac{1}{V_{gut}} \left(Q_{gut} c_{art} - Q_{gut} \frac{c_{gut}}{P_{gut:B}} + input_{GI} \right) \quad \text{(S13)}$$

$input_{GI}$ is the mass flow representing the drug absorption from the gastrointestinal (GI) tract, defined in equation (S19).

$P_{T:B}$ values were calculated *in*-silico using the method of Rodgers & Rowland (14,15). The Rodgers & Rowland method allows to derive the tissue to plasma unbound partition coefficient, $P_{T:P,u}$. The values of $P_{T:B}$ can be obtained from $P_{T:P,u}$ as follows:

$$P_{T:B} = \frac{P_{T:P,u}}{BP} f_{up} \quad \text{(S14)}$$

where f_{up} is the fraction of drug unbound in plasma and BP is the blood to plasma ratio.

Concerning the metabolism, the E_R was directly calculated using the *in vivo* plasma concentration data as per (2):

$$E_R = \frac{CL_p}{Q_{liv}} \quad \text{(S15)}$$

where CL_p is the plasma clearance obtained by Non Compartmental Analysis.

1.3 GI absorption

When the drug is administered via the pulmonary route, a portion of the dose gets swallowed. For this reason, we added a model describing the GI absorption. In particular, we adopted a model derived from the Compartmental Absorption and Transit (CAT) model (16). Here, we considered the GI system divided in 9 sections: one section for the stomach, seven sections for the small intestine and one for the large intestine. Each GI section is composed of a compartment in which the drug is in the solid form and by a compartment in which the drug is dissolved in the physiological fluids. The solid drug can dissolve or transit in the subsequent section, while the dissolved drug can get absorbed or transit in the subsequent section too. From the large intestine the drug can be excreted. The absorption was supposed to happen only in the small intestine.

The mass balance equations for the stomach compartments are reported below.

$$\frac{da_{st,und}}{dt} = -k_{t,st}a_{st,und} - k_{d,st}a_{st,und}$$
$$\frac{da_{st,diss}}{dt} = +k_{d,st}a_{st,und} - k_{t,st}a_{st,diss} \quad \text{(S16)}$$

$a_{st,und}$ is the stomach undissolved drug amount, $k_{t,st}$ the stomach transit constant and $k_{d,st}$ the dissolution rate constant.

The equations for the first small intestine segment are:

$$\frac{da_{1,und}}{dt} = +k_{t,st}a_{st,und} - k_{t,int}a_{1,und} - k_{d,1}a_{1,und}$$
$$\frac{da_{1,diss}}{dt} = +k_{t,st}a_{st,diss} - k_{t,int}a_{1,diss} + k_{d,1}a_{1,und} - k_a a_{1,diss} \quad \text{(S17)}$$

where $a_{1,und}$ and $a_{1,diss}$ are the undissolved and dissolved amount in the first small intestine segment, respectively. $k_{t,int}$ is the transit constant. The transit constants have been calculated as $1/MRT$, where MRT is the mean transit time. For each small intestine segment, the MRT has been calculated dividing the literature total small intestine MRT for the total number of segments (that in this model it is assumed to be 7).

The equations for all the other small intestine segments are reported below (with $i = 2 \dots 7$).

$$\frac{da_{i,und}}{dt} = +k_{t,int}a_{i-1,und} - k_{t,int}a_{i,und} - k_{d,i}a_{i,und}$$
$$\frac{da_{i,diss}}{dt} = +k_{t,int}a_{i-1,diss} - k_{t,int}a_{i,diss} + k_{d,i}a_{i,und} - k_a a_{i,diss} \quad \text{(S18)}$$

The amount of dissolved drug that gets absorbed through the gut wall is supposed to directly enter the gut compartment of the PBPK model. $input_{GI}$ in equation (S13) was calculated as follows.

$$input_{GI} = \sum_{i=1}^{7} k_a \cdot a_{i,diss} \quad \text{(S19)}$$

For the last segment, representing the large intestine, the equations of the model are the following:

$$\frac{da_{li,und}}{dt} = +k_{t,8}a_{8,und} - k_{t,li}a_{li,und} - k_{d,li}a_{li,und}$$
$$\frac{da_{li,diss}}{dt} = +k_{t,8}a_{8,diss} - k_{t,li}a_{li,diss} + k_{d,li}a_{li,und} \quad \text{(S20)}$$

$a_{li,und}$ and $a_{li,diss}$ are the undissolved and dissolved amounts in the large intestin. $k_{t,li}$ is the large intestine transit constant.

Similarly to what was done for modelling the dissolution in the ELF, k_d was calculated by using the Noyes-Whitney model, as described in section 1.1. Here the solubility has been calculated taking into account the changes in the pH across the GI sections. The dependency of the solubility on the pH was described

according to the Henderson-Hasselbalch equations.
The drug absorption was modelled as a first order process with the absorption rate constants k_a, calculated starting from the measure of the *in-vitro* apparent drug permeability across the Caco-2 cells layer (16).

$$k_a = P_{eff} \cdot ASF \tag{S21}$$

P_{eff} is the effective permeability derived from the apparent permeability measured on the Caco-2 cells and ASF is the absorption scaling factor, assumed to be equal to $2/r_{int}$, with r_{int} the mean intestinal radius.

The value of human effective permeability, $P_{eff,human}$, was calculated as (17):

$$\log(P_{eff,human}) = 0.4926 \cdot \log(P_{app,pH7.4}) - 0.1454 \tag{S22}$$

and then the effective permeability in rats, $P_{eff,rat}$, was obtained with the following relationship (17).

$$P_{eff,human} = 3.6 \cdot P_{eff,rat} + 0.03 \tag{S23}$$

2 Drug distribution in the lungs

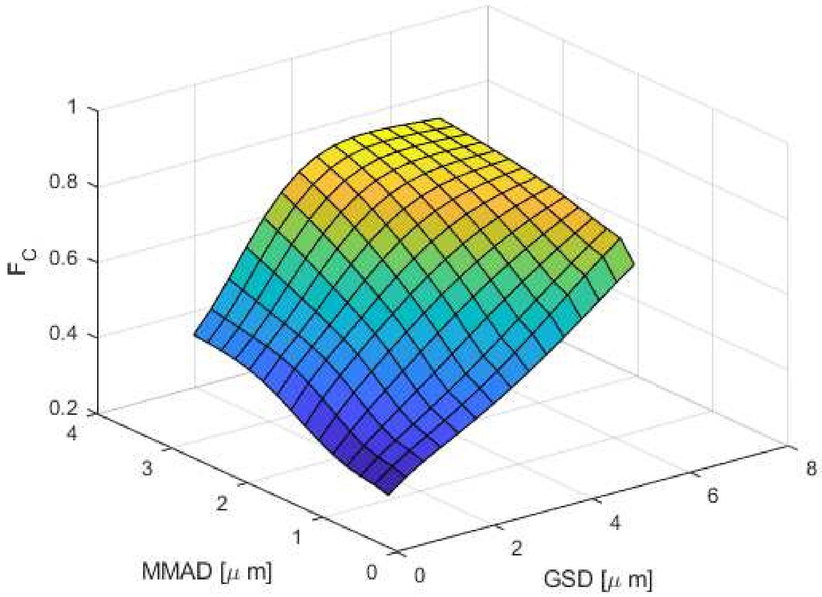

Figure S1: *F_C values calculated from $MMAD$ and GSD by using MPPD.*

The central fraction (F_C) of the total inhaled dosed was calculate from the values of MMAD and GSD. The software MPPD2.11 (18,19) was used for this purpose, with the hypotheses of drug true density (ρ) equal to 1 g/cm^3. In particular, F_C was derived as in equation (S1).

$$F_C = \frac{F_{central,MPPD}}{F_{central,MPPD} + F_{peripheral,MPPD}} \quad \text{(S1)}$$

$F_{central,MPPD}$ and $F_{peripheral,MPPD}$ are the central fraction and peripheral pulmonary fraction predicted by MPPD simulating an intratracheal delivery. This was done because, using the precise inhale technology (20) for compound administration in rats, a complete inhalation of the dose can be supposed, hence the fraction exhaled and the extrathoracic deposition predicted has been neglected. During GSA, the model is repetitively evaluated for different parameter sets. Calling MPPD at each model evaluation for computing F_C would not be an optimal solution from a computational point of view. To reduce the computational cost, we pre-calculated the values of F_C with MPPD for a 15x15 grid of MMAD and GSD values, thus, generating the surface reported in **Figure S1**. F_C was then calculated at each iteration for given values of MMAD and GSD through linear interpolation of this surface.

The impact of ρ on F_C calculation with MPPD was assessed by calculating F_C for the minimum and maximum values of ρ in **Table I** of the main text (0.5 and 1.5 mg/mL). The maximum observed fold change between F_C for $\rho = 0.5\, mg/mL$ and $\rho = 1.5\, mg/mL$ was approximately 2.5%. Given its little impact, ρ value was always considered equal to 1 g/cm^3 during F_C computation.

3 *In vitro* permeability model

Passive and the active permeabilities were estimated from the measurements obtained in the *in vitro* Calu3 permeability assay, by fitting a model describing the major *in vitro* transport processes.

Due to the capability of reaching high trans epithelial electric resistance values and for their transporter expression similar to those of the respiratory tissues, Calu3 cell lines are commonly used to assess the permeability of inhaled drugs (3). This type of cell is robust and easy to culture, so, it can be used for routine screening purposes. Furthermore, good correlation between the permeability values and the *in vivo* drug absorption in drug lung was reported (4,5). During the experiment, cells are exposed to a liquid medium in both the apical and the basolateral surface. A 10 µM compound solution is added in the liquid on one side (e.g., apical) and the concentration is monitored in the opposite side (e.g., basolateral). The data allow to obtain the apparent permeability P_{app} (for the Apical-Basolateral and Basolateral-Apical directions), typically calculated with the following equation:

$$P_{app} = \frac{\Delta Q/\Delta t}{C_o A}$$

where ΔQ is the change in the amount of drug in the receiving fluid in the Δt interval, C_o is the initial concentration in the medium in which the compound is administered, A is the cell layer surface area. The obtained value is the net result of passive and active transport.

To distinguish between passive and active transport, in Grandoni et al. (1) a mechanistic model of the *in vitro* Calu3 permeability assay was built. The model is composed by three compartments: the apical media, the cells and the basolateral media. It is assumed that the main fluxes in the system are due to the passive transcellular bidirectional transport between the cells and the fluids and to the monodirectional efflux from the tissue to the apical fluid. The model is reported in equation system (S2).

$$\begin{aligned}
\frac{dc_{AF}}{dt} &= \frac{1}{V_{AF}}(-P_p\, A\, c_{AF} + P_p\, A\, c_C\, f_{u,t} + P_a\, A\, c_C\, f_{u,t}) \\
\frac{dc_C}{dt} &= \frac{1}{V_C}(-2P_p\, A\, c_C\, f_{u,t} - P_a\, A\, c_C\, f_{u,t} + P_p\, A\, c_{AF} + P_p\, A\, c_{BF}) \\
\frac{dc_{BF}}{dt} &= \frac{1}{V_{BF}}(-P_p\, A\, c_{BF} + P_p\, A\, c_C\, f_{u,t})
\end{aligned} \qquad \text{(S2)}$$

c_{AF}, c_{BF} and c_C are the drug concentrations in the apical and basolateral fluid and in the cell layer, respectively; V_{AF}, V_{BF} and V_C are the apical and basolateral fluid and cells volumes, respectively; A is the area of the cell culture, P_p and P_a are the passive and active permeabilities. P_p and P_a values are estimated by simultaneously fitting the basolateral media concentration data obtained with the apical-basolateral experiment and the apical medium concentration data after obtained with the basolateral-apical permeability experiment.

4 PBPK model parameters

4.1 PBPK volumes, fluxes and organ surfaces

In (21), the different organ weights are reported as a percentage of the total body weight (relative organ weight); to obtain organ volumes from these values for a typical subject with a body weight of 250 g was considered. Moreover, it was assumed that tissues have the same density of water. To estimate the percentage of adipose tissue in the total body weight, the following relationship reported for Sprague-Dawley rats has been used:

$$\%\, Adipose\ Tissue = 0.199 \cdot BW + 1.664$$

where BW is the body weight, in grams.

The volume of the rest of the body compartment was calculated assuming that the tissues included in the model represent the 85% of the total body weight (BW) (22). Hence, its weight has been calculated subtracting the sum of all the other weights from the 85% of BW. The volume of blood was taken from the work of Davies et. al., (23), in which the blood and plasma volumes for a rat of 250 g of body weight are reported. In the same work, the repartition between arterial and venous blood was reported, a 75% vs 25% split was suggested for arterial and venous blood, respectively. These values were adopted in this model. The volumes of the extravascular and vascular compartments for the tissues with a permeability-limited kinetics were calculated from the volume fractions of vascular and interstitial spaces, that can be found in (24). Here, the rest of the body compartment was supposed to be composed by bones and skin. Here, the vascular compartment includes also the interstitial space.

Concerning the blood flows, in (21) the regional flow distribution in different organs as a percentage of the cardiac output is reported. The cardiac output (Q_{CO}, expressed in L/h) has been calculated for a standard rat of 250 g using the allometric equation reported in (21).

$$Q_{CO} = 15 \cdot (BW)^{0.74}$$

In the previous equation, BW is expressed in kg. The value of the liver flux was calculated as sum of the gut, spleen and hepatic artery fluxes.

The percentage of Q_{CO} directed to the gut and the spleen was taken from (25).

According to the authors' knowledge, the organ surfaces needed to compute the permeability-surface product (PS) for the organs whose distribution was considered permeability limited (muscle and rest of the body) are not present in the literature. The only exception found was for the lungs, where volumes and surfaces of the tracheobronchial and alveolar regions are available (central and peripheral lung region, respectively) (26). Hence, surfaces for other organs or tissues were obtained by scaling the surface area of the tracheobronchial region through the following formula (2):

$$S_T = S_{TB}\left(\frac{V_{ev,T}}{V_{ev,C}}\right)^{\frac{2}{3}}$$

where S_T and $V_{ev,T}$ are the surface and the EV volume of the tissue T of interest, respectively; analogously, S_{TB} and $V_{ev,C}$ are the tracheobronchial surface and EV volume, respectively.

Table SI: ***PBPK organs volumes and blood flows for a 250 g rat***

**Organs**	**Volumes** $[\boldsymbol{mL}]$ [a]	**Blood flows** $[\boldsymbol{mL/min}]$ [b]
Brain	1.43	1.79
Gut	6.75	11.92
Spleen	0.5	0.8
Liver	9.15	14.6
Muscles	101.8	24.91
Adipose	16.6	6.27
Heart	0.83	4.39
Kidney	1.83	12.64
Rest of the body	72.36	23.1
Venous blood	10.12	
Arterial blood	3.38	

[a] (21–23)
[b] (21-25)

4.2 Pulmonary & gastrointestinal absorption model parameters

Table SII: ***lung physiological parameters***

Parameters	Central lung	Peripheral lung	Unit	Reference
Surface area	276.4	3.27	dm^2/kg	(26)
Lining fluid volume	163.6	193.5	$\mu L/kg$	(26)
Tissue volumes[a]	1.01	0.24	mL	(21,26)
Blood flows[b]	89.61	1.88	mL/min	(21,26)
Proportion of extravascular tissue[c]	0.45	0.45		(2,24)

[a] The tissue volumes were obtained multiplying the total lung volume, from (21), for the proportions reported in (26).
[b] The blood flows were obtained multiplying the CO, from (21), for the proportions reported in (26).
[c] The proportions of vascular and extravascular tissue in the central and peripheral lung regions were obtained from (24), as shown in (2). Both the values were assumed to be the same.

Table SIII: ***rat physiological GI parameters***

GI section	Volumes $[mL]$ [a]	pH [a]	k_t $[s^{-1}]$
Stomach	3	3	0.0017
Small intestine, section 1	0.6	7.1	0.0013
Small intestine, section 2	0.66	7.3	0.0013
Small intestine, section 3	0.66	7.5	0.0013
Small intestine, section 4	0.41	7.7	0.0013
Small intestine, section 5	0.41	7.9	0.0013
Small intestine, section 6	0.41	8	0.0013
Small intestine, section 7	0.41	7.4	0.0013
Large intestine	3	7.6	0.000073

[a] (13,27)

Table SIV: ***parameters used to calculate the dissolution constant***

Parameters	Value	Units	Reference
T: absolute body temperature	$310.15\ (37)$	$K\ (°C)$	(28)
η_w: dynamic viscosity of water at 37°C [a]	$6.91 \cdot 10^{-4}$	$Pa \cdot s$	
η_{slf}: dynamic viscosity of SLF [b]	$100 \cdot 10^{-4}$	$Pa \cdot s$	(29)
k_b: Boltzmann constant	1.3806504	$10^{-23} J/K$	
N_A: Avogadro's number	6.02214179	$10^{23} mol^{-1}$	

[a] Used to calculate k_d for the drug dissolution in the intestinal lumen

[b] Used to calculate k_d for the drug dissolution in the extracellular lining fluid

5 PBPK model performances

Below the PBPK model performances of the nine compounds belonging to the Chiesi Farmaceutici portfolio considered in this analysis are reported (1).

5.1 PBPK prediction performances on lung metrics

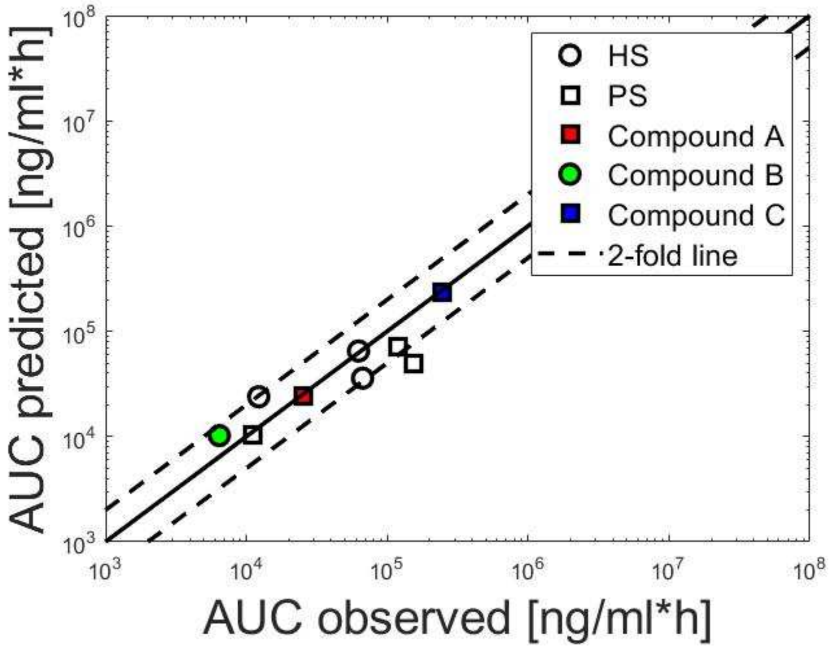

Figure S2*: predicted versus observed whole lung AUC.*

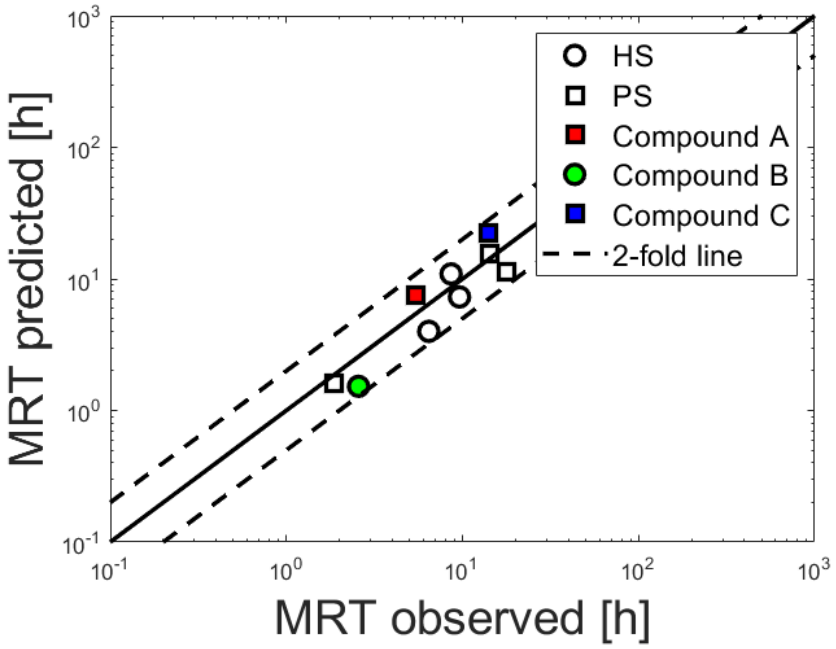

Figure S3*: predicted versus observed whole lung MRT.*

5.2 PBPK prediction performances on plasma metrics

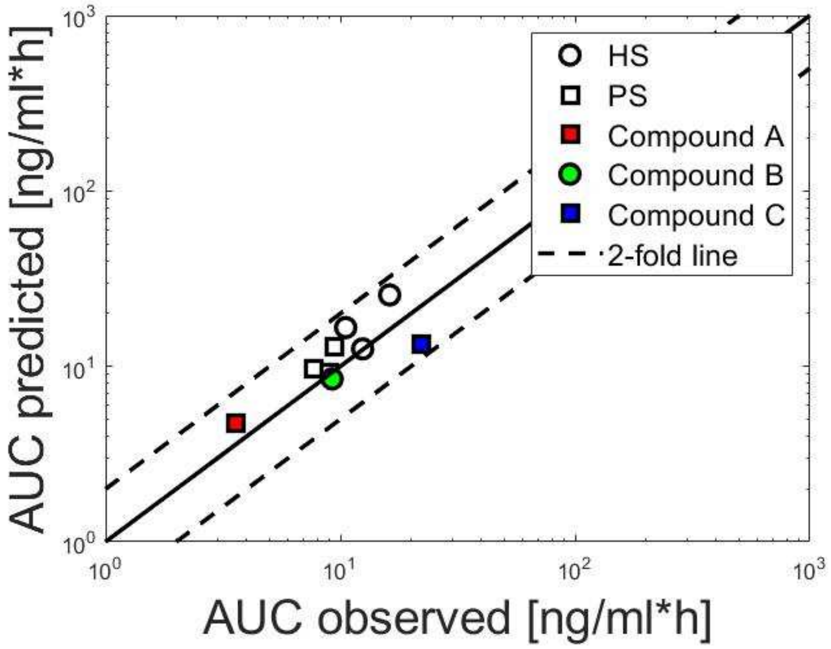

***Figure S4**: predicted versus observed plasma AUC.*

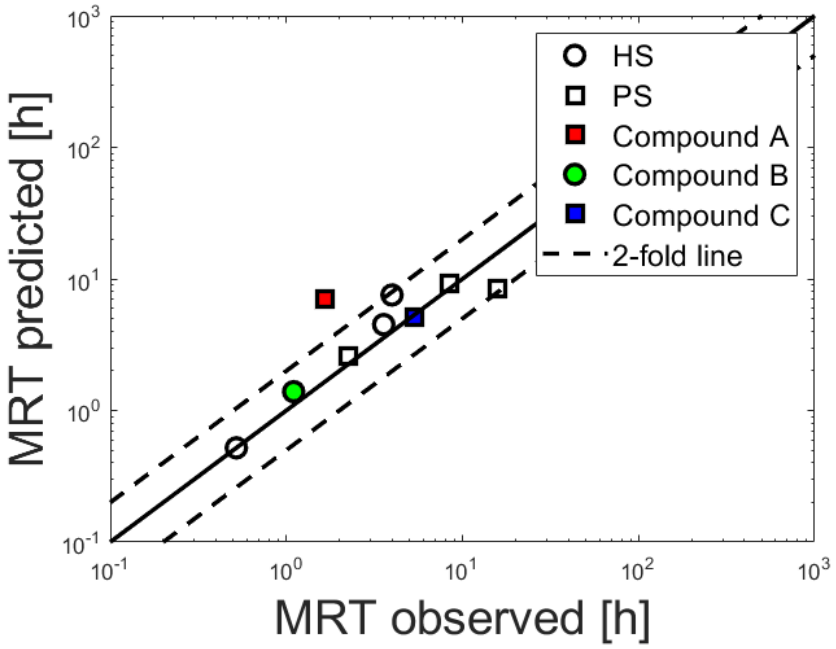

***Figure S5**: predicted versus observed plasma MRT.*

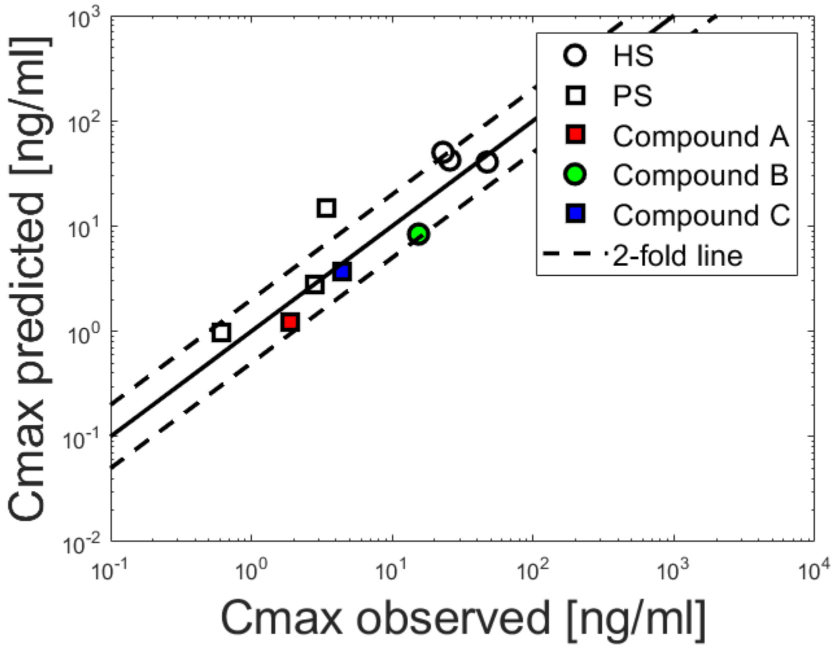

***Figure S6**: predicted versus observed plasma C_{max}.*

6 Histograms of model outputs for Global Sensitivity Analysis

6.1 *Inter-compound GSA*

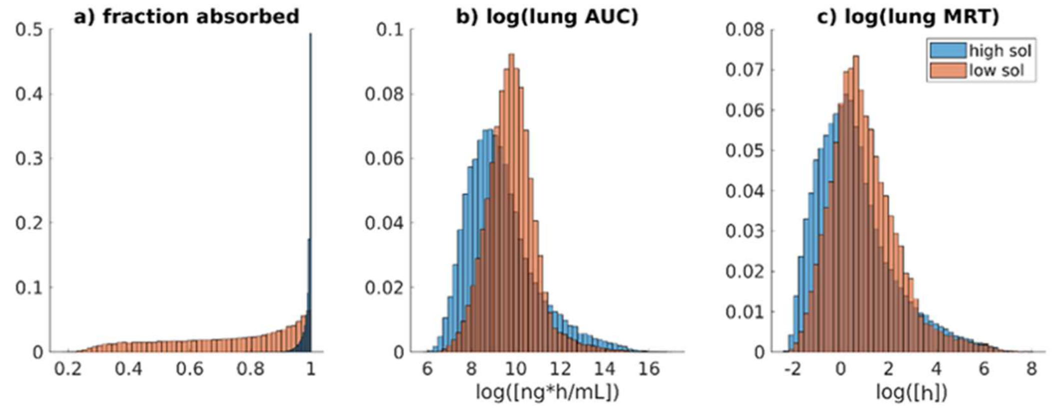

Figure S7: Inter-compounds variability of (a) fraction absorbed, (b) logarithm of whole lung AUC and (c) logarithm of whole lung MRT for highly and poorly soluble compounds

6.2 *Intra-compound GSA*

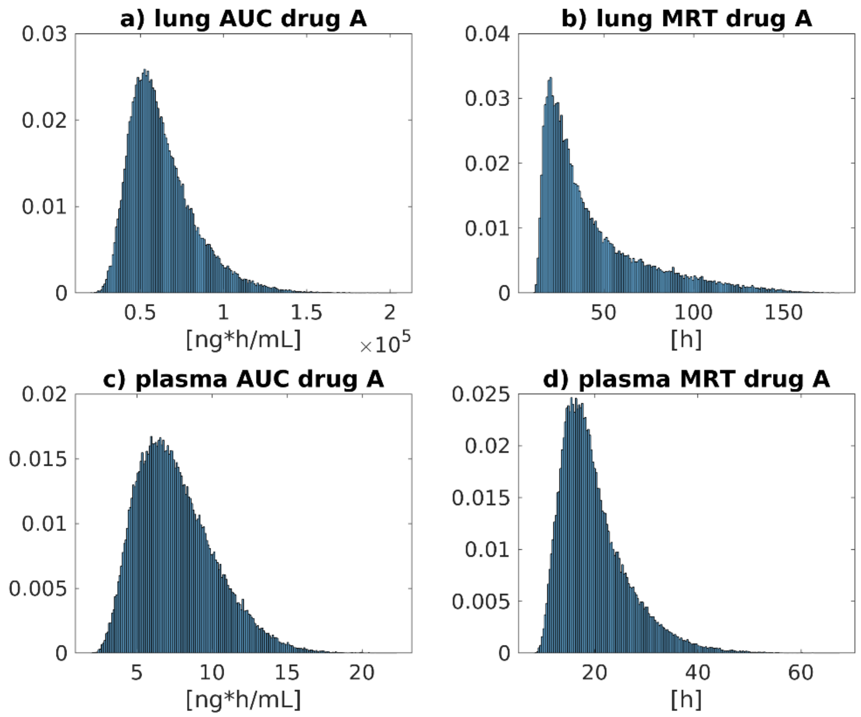

Figure S8: Intra-compound variability of (a) whole lung AUC, (b) whole lung MRT, (c) plasma AUC and (d) plasma MRT for compound A.

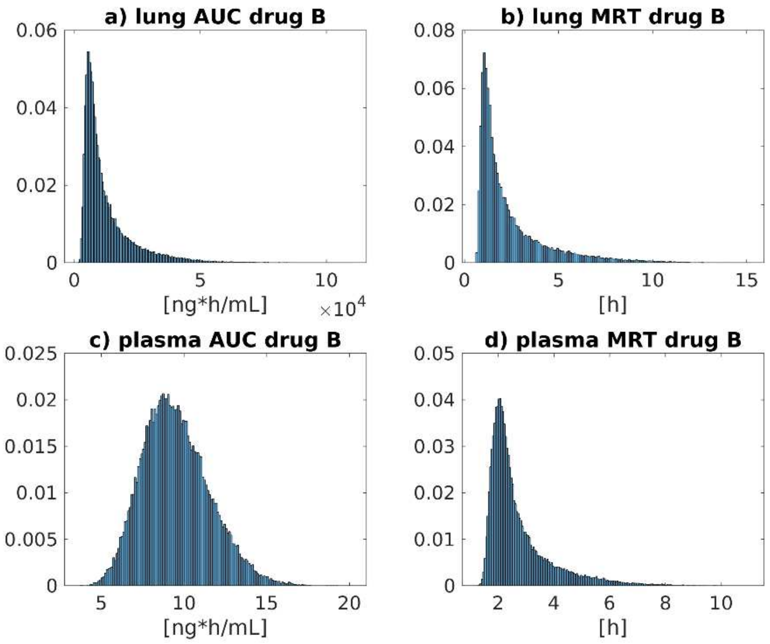

Figure S9: Intra-compound variability of (a) whole lung AUC, (b) whole lung MRT, (c) plasma AUC and (d) plasma MRT for compound B

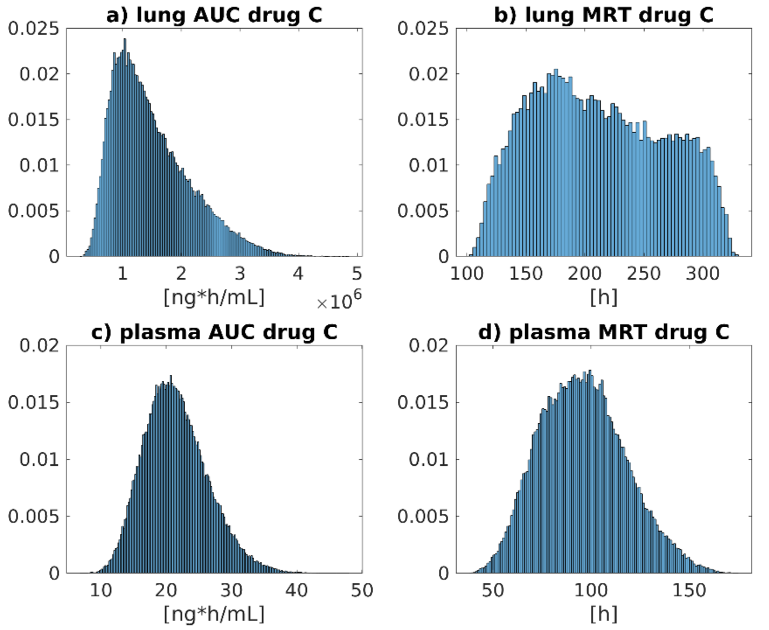

Figure S10: Intra-compound variability of (a) whole lung AUC, (b) whole lung MRT, (c) plasma AUC and (d) plasma MRT for compound C.

7 References

1. Grandoni S, Cesari N, Melillo N, Brogin G, Puccini P, Magni P. Development and evaluation of a PBPK model to study the pharmacokinetics of inhaled drugs in rats. In: PAGE 28, Abstr 9047. Stockholm, Sweden.; 2019. Available from: www.page-meeting.org/?abstract=9047

2. Grandoni S, Cesari N, Brogin G, Puccini P, Magni P. Building in-house PBPK modelling tools for oral drug administration from literature information. ADMET and DMPK. 2019 Feb 23;7(1):4–21.

3. Meindl C, Stranzinger S, Dzidic N, Salar-Behzadi S, Mohr S, Zimmer A, et al. Permeation of Therapeutic Drugs in Different Formulations across the Airway Epithelium In Vitro. PLOS ONE. 2015 Aug 14;10(8):e0135690.

4. Bosquillon C, Madlova M, Patel N, Clear N, Forbes B. A Comparison of Drug Transport in Pulmonary Absorption Models: Isolated Perfused rat Lungs, Respiratory Epithelial Cell Lines and Primary Cell Culture. Pharm Res. 2017 Dec 1;34(12):2532–40.

5. Mathias NR, Timoszyk J, Stetsko PI, Megill JR, Smith RL, Wall DA. Permeability Characteristics of Calu-3 Human Bronchial Epithelial Cells: In Vitro - In Vivo Correlation to Predict Lung Absorption in Rats. Journal of Drug Targeting. 2002 Jan 1;10(1):31–40.

6. Noyes AA, Whitney WR. The rate of solution of solid substances in their own solutions. Journal of the American Chemical Society. 1897 Dec;19(12):930–4.

7. Hinds WC. Uniform Particle Motion. In: Aerosol technology Properties, Behavior, and Measurement of Airborne Particles. Second Edition. John Wiley & Sons Inc.; 1999. p. 42–74.

8. Pepin XJH, Flanagan TR, Holt DJ, Eidelman A, Treacy D, Rowlings CE. Justification of Drug Product Dissolution Rate and Drug Substance Particle Size Specifications Based on Absorption PBPK Modeling for Lesinurad Immediate Release Tablets. Molecular Pharmaceutics. 2016 Jul 20;13(9):3256–69.

9. Hintz RJ, Johnson KC. The effect of particle size distribution on dissolution rate and oral absorption. International Journal of Pharmaceutics. 1989 Apr;51(1):9–17.

10. Germani M, Crivori P, Rocchetti M, Burton PS, Wilson AGE, Smith ME, et al. Evaluation of a basic physiologically based pharmacokinetic model for simulating the first-time-in-animal study. European Journal of Pharmaceutical Sciences. 2007 Jul 1;31(3):190–201.

11. Poulin P, Theil F-P. Prediction of Pharmacokinetics Prior to In Vivo Studies. II. Generic Physiologically Based Pharmacokinetic Models of Drug Disposition. JPharmSci. 2002 May 1;91(5):1358–70.

12. Jeong Y-S, Yim C-S, Ryu H-M, Noh C-K, Song Y-K, Chung S-J. Estimation of the minimum permeability coefficient in rats for perfusion-limited tissue distribution in whole-body physiologically-based pharmacokinetics. European Journal of Pharmaceutics and Biopharmaceutics. 2017 Jun 1;115:1–17.

13. Peters SA. Physiologically-Based Pharmacokinetic (PBPK) Modeling and Simulations: Principles, Methods, and Applications in the Pharmaceutical Industry. Hoboken, New Jersey: John Wiley & Sons, Inc.; 2012.

14. Rodgers T, Leahy D, Rowland M. Physiologically Based Pharmacokinetic Modeling 1: Predicting the Tissue Distribution of Moderate-to-Strong Bases. Journal of Pharmaceutical Sciences. 2005 Jun;94(6):1259–1276.

15. Rodgers T, Rowland M. Physiologically based pharmacokinetic modelling 2: Predicting the tissue distribution of acids, very weak bases, neutrals and zwitterions. Journal of Pharmaceutical Sciences. 2006 Jun;95(6):1238–1257.

16. Yu LX, Amidon GL. A compartmental absorption and transit model for estimating oral drug absorption. International Journal of Pharmaceutics. 1999 Sep;186(2):119–125.

17. Yang J, Jamei M, Rowland-Yeo K, Tucker G, Rostami-Hodjegan A. Prediction of Intestinal First-Pass Drug Metabolism. Current Drug Metabolism. 2007;8(7).

18. Anjilvel S, Asgharian B. A Multiple-Path Model of Particle Deposition in the Rat Lung. Fundamental and Applied Toxicology. 1995 Nov 1;28(1):41–50.

19. National Institute for Public Health and the Environment (RIVM). Multiple Path Particle Dosimetry Model (MPPD v 1.0): A Model for Human and Rat Airway Particle Dosimetry. Bilthoven, The Netherlands. Bilthoven, The Netherlands.: RIVA Report 650010030.; 2002.

20. Gerde P, Ewing P, Låstbom L, Ryrfeldt Å, Waher J, Lidén G. A Novel Method to Aerosolize Powder for Short Inhalation Exposures at High Concentrations: Isolated Rat Lungs Exposed to Respirable Diesel Soot. Inhalation Toxicology. 2004 Jan 1;16(1):45–52.

21. Brown RP, Delp MD, Lindstedt SL, Rhomberg LR, Beliles RP. Physiological Parameter Values for Physiologically Based Pharmacokinetic Models. Toxicol Ind Health. 1997 Jul 1;13(4):407–84.

22. U.S. EPA. Approaches For the Application of Physiologically Based Pharmacokinetic (PBPK) Models and Supporting Data In Risk Assessment (Final Report). Washington, D.C.: U.S. Environmental Protection Agency; 2006. Report No.: EPA/600/R-05/043F.

23. Davies B, Morris T. Physiological Parameters in Laboratory Animals and Humans. Pharm Res. 1993 Jul 1;10(7):1093–5.

24. Kawai R, Mathew D, Tanaka C, Rowland M. Physiologically Based Pharmacokinetics of Cyclosporine A: Extension to Tissue Distribution Kinetics in Rats and Scale-up to Human. J Pharmacol Exp Ther. 1998 Nov 1;287(2):457–68.

25. Delp MD, Manning RO, Bruckner JV, Armstrong RB. Distribution of cardiac output during diurnal changes of activity in rats. American Journal of Physiology-Heart and Circulatory Physiology. 1991 Nov 1;261(5):H1487–93.

26. Boger E, Evans N, Chappell M, Lundqvist A, Ewing P, Wigenborg A, et al. Systems Pharmacology Approach for Prediction of Pulmonary and Systemic Pharmacokinetics and

Receptor Occupancy of Inhaled Drugs. CPT: Pharmacometrics & Systems Pharmacology. 2016;5(4):201–10.

27. Peters SA. Evaluation of a Generic Physiologically Based Pharmacokinetic Model for Lineshape Analysis. Clin Pharmacokinet. 2008 Apr 1;47(4):261–75.

28. Lillie LE, Temple NJ, Florence LZ. Reference values for young normal Sprague-Dawley rats: weight gain, hematology and clinical chemistry. Hum Exp Toxicol. 1996 Aug 1;15(8):612–6.

29. Salomone F, Gallem T, Hetzer U. Pharmaceutical formulation comprising pulmonary surfactant for administration by nebulization. WO2019115802A1, 2019 [cited 2020 Jun 29]. Available from: https://patents.google.com/patent/WO2019115802A1/en
